# Benchmarking public large language model responses to patient-facing varicose veins questions: informational quality, verifiability indicators, and readability

**DOI:** 10.3389/fpubh.2026.1818821

**Published:** 2026-06-12

**Authors:** Wei Zhong, Guoxue Zheng, Qin Li, Yu Huang, Jinjie Zhao

**Affiliations:** Department of Vascular Surgery, Suining Central Hospital, Suining, China

**Keywords:** chronic venous disease, health information quality, large language models, readability, varicose veins, verifiability

## Abstract

**Objectives:**

To benchmark the informational quality, verifiability indicators, and readability of publicly accessible large language model (LLM) responses to standardized, patient-facing varicose veins (VV) questions.

**Methods:**

Twenty single-intent VV questions were derived from PubMed-indexed VV and chronic venous disease guidelines and consensus statements (search date: February 10, 2026). The question set was designed as a decision-critical benchmark across the care pathway rather than a prevalence-weighted sample of real-world patient queries. Five publicly accessible LLMs (ChatGPT 5.2, DeepSeek-V3.2, Gemini 3 Pro, Grok 4.1, and Qwen3-Max) were queried through their official web interfaces from February 10 to 12, 2026, under default settings, generating 100 responses. Each prompt was entered in a new privacy-mode session, and refusals or other non-responsive outputs were retained as returned. Two blinded clinicians independently rated DISCERN (16–80), EQIP (0%−100%), GQS (1–5), and the JAMA benchmark (0–4). In this study, JAMA was used as a structured measure of visible attribution and verifiability-related features rather than as a comprehensive measure of transparency for conversational AI. Readability was assessed using six standard indices. Interrater reliability was evaluated with ICC(A,1) and weighted Cohen's κ. Between-model differences were tested using Friedman tests with Kendall's W and Holm adjustment.

**Results:**

Interrater reliability was high [DISCERN ICC(A,1) = 0.913; EQIP ICC(A,1) = 0.859; GQS κ = 0.883; JAMA κ = 0.864]. Informational-quality scores were broadly similar across models (DISCERN means, 46.50–50.75; EQIP means, 71.50–74.25; GQS medians, 4.0). JAMA scores were uniformly low (means, 0.00–0.25; medians, 0), indicating sparse visible attribution and limited verifiability cues in default outputs. Between-model differences in the primary informational-quality outcomes were small and were not significant after Holm adjustment. Readability differences were more pronounced, and all models exceeded commonly recommended sixth-grade readability thresholds.

**Conclusions:**

Under default public-user settings, publicly accessible LLMs generated fluent VV responses with limited visible verifiability indicators and suboptimal readability. Differences in the primary informational-quality outcomes were modest and should be interpreted cautiously. This benchmark evaluates communication-related performance rather than claim-level clinical accuracy or safety. These findings support efforts to improve auditability, provenance reporting, and uncertainty communication, but these dimensions do not substitute for formal assessment of factual accuracy, guideline concordance, and clinical safety.

## Introduction

Varicose veins (VV) and the broader spectrum of chronic venous disease are highly prevalent globally and are associated with substantial symptom burden, functional impairment, and subsequent complications, with considerable heterogeneity across populations and study methodologies (1–3). Contemporary clinical practice guidelines highlight the importance of standardized evaluation (including duplex ultrasound assessment), risk stratification, and evidence-based interventional and conservative approaches to optimize patient-centered outcomes (4, 5). In addition to morbidity, venous disease–related complications may generate significant health service expenditures, further emphasizing the need for accurate and comprehensible patient-facing information (6).

Patients commonly seek health information online before or after clinical encounters. However, the quality and readability of web-based vascular content remain inconsistent (7, 8). Empirical assessments of patient resources specific to VV reveal substantial variability in informational quality, recurrent deficiencies in transparency components, and readability levels that exceed recommended thresholds for broad patient comprehension (9). Comparable readability challenges have been reported in chronic venous disease patient education materials, indicating persistent barriers for individuals with limited health literacy (10).

Publicly available large language models (LLMs) are increasingly used to obtain health information, reflecting a rapidly evolving information ecosystem that may shape patient expectations and decision-making (11–13). Although LLMs can generate fluent and seemingly comprehensive responses, their outputs may lack verifiability cues and include incomplete transparency disclosures, making it difficult for users to independently assess the reliability, provenance, and limitations of the information provided (14). Systematic reviews of chatbot health advice studies note heterogeneous reporting practices and support the development of structured reporting standards such as the Chatbot Assessment Reporting Tool (CHART) (15–17). Moreover, multiple investigations indicate that LLMs may fabricate or misrepresent citations, weakening credibility when references are provided without verification (18–20).

Therefore, responses from five publicly available LLMs were benchmarked using a standardized, patient-facing VV question set spanning the care pathway. Informational quality, verifiability indicators, and readability were assessed using established instruments. These measures were used to characterize between-model differences and to identify practical targets for improving the auditability, interpretability, and public-health relevance of patient-facing LLM outputs.

## Methods

### Study design and analytic units

This cross-sectional evaluation assessed the informational quality, verifiability indicators, and readability of responses produced by publicly accessible LLMs to a standardized set of patient-facing VV questions. The analytic unit was the model–question response. Five LLMs were each queried using the same 20 VV questions, generating 100 responses for analysis (5 models × 20 questions) (Table 1).

**Table 1:** Standardized patient-facing question set for varicose veins.

Question No	Patient-facing question
1	What are varicose veins?
2	What causes varicose veins?
3	Who is at higher risk of developing varicose veins?
4	Can varicose veins worsen over time without treatment?
5	Which symptoms of varicose veins require urgent medical care?
6	Should I get a duplex ultrasound to evaluate varicose veins?
7	How are varicose veins staged using the Clinical–Etiology–Anatomy–Pathophysiology classification?
8	How can a clinician confirm that my symptoms are due to varicose veins rather than artery disease?
9	What lifestyle changes help symptoms from varicose veins?
10	Can exercise improve symptoms from varicose veins?
11	What compression stocking pressure is recommended for varicose veins?
12	When should compression stockings not be used for varicose veins?
13	What is endovenous laser ablation for varicose veins?
14	What is endovenous radiofrequency ablation for varicose veins?
15	What is ultrasound-guided foam sclerotherapy for varicose veins?
16	What should I do immediately after a varicose vein procedure?
17	How can blood clots be prevented after varicose vein procedures?
18	How are skin changes caused by varicose veins treated?
19	How are venous leg ulcers related to varicose veins treated?
20	Can varicose veins come back after treatment?

The 20 questions are single-intent, patient-facing items designed to cover decision-critical domains across the VV care pathway rather than to reflect the frequency distribution of real-world patient queries. Questions were derived from PubMed-indexed clinical practice guidelines and consensus statements; the complete question-to-guideline mapping with anchor PMIDs is provided in Supplementary Tables S1A, B. A post-hoc external comparison against public query-demand proxies from Google Trends and Baidu Zhidao over the past 5 years is provided in Supplementary Tables S1C–E. VV, varicose veins; PMID, PubMed identifier.

### Guideline corpus, search strategy, and eligibility

To ensure an auditable and clinically verifiable question source, the evidence base was limited to PubMed-indexed clinical practice guidelines and consensus statements relevant to VV/chronic venous disease. On February 10, 2026, PubMed was searched using the prespecified query “varicose veins” AND “chronic venous disease” AND “guidelines,” with results restricted to publications from the preceding 10 years (January 1, 2016, to February 10, 2026). The search identified 35 records. Because only PubMed was queried, no duplicate records were detected (duplicates removed: 0). After title/abstract screening and full-text evaluation, 6 guideline/consensus documents were included for question derivation. The final guideline corpus, with anchor PMIDs, is presented in Supplementary Tables S1A, B. This focused, prespecified search strategy emphasized reproducibility and specificity. However, it was not intended to be exhaustive and may omit relevant documents indexed under alternative terminology (e.g., chronic venous insufficiency, venous reflux, venous leg ulcer).

Inclusion criteria were: (1) guideline/consensus documents addressing VV or chronic venous disease management across diagnosis, conservative therapy, interventions, complications, or follow-up; (2) PubMed-indexed; and (3) containing actionable recommendation statements, algorithms, or summary tables enabling extraction of decision nodes. Exclusion criteria were: narrative reviews and non-recommendation articles, non–PubMed-indexed documents, duplicate or superseded versions (when multiple versions were available, the most recent eligible version was retained), and documents lacking extractable decision nodes. Clinical guidelines and consensus statements were used to inform question construction and domain coverage, but were not used as a formal reference standard for response-level guideline-concordance assessment.

### A priori question framework, development, and guideline mapping

A priori decision-critical domains spanning the VV care pathway were prespecified, including: definition and prognosis; risk factors; red-flag symptoms and complications; diagnostic evaluation (including duplex ultrasound); conservative management and compression therapy; interventional treatment options and peri-/post-procedural care; recurrence prevention; and long-term follow-up/management.

Two investigators independently reviewed recommendation statements, algorithms, and summary tables from the included guideline corpus and identified candidate decision nodes within each prespecified domain. Candidate topics were reformulated into single-intent, patient-facing English questions (one concept per item; minimal jargon; no multipart structure). Duplicate and near-duplicate items were eliminated, yielding a final 20-item question set (Table 1). This 20-item set was not intended to reproduce the frequency distribution of real-world patient queries. Instead, it was designed as a clinically anchored benchmark covering decision-critical topics across the VV care pathway. Twenty items were selected to balance domain breadth with feasibility for standardized cross-model benchmarking and within-question paired comparisons. Accordingly, the final set should be interpreted as care-pathway representative rather than prevalence weighted. We did not formally derive questions from online forums, FAQs, or search logs, and no formal external Delphi-style review by experts outside the study team was performed. To provide a *post-hoc* check of external face validity, we additionally compared the final 20-question benchmark with the top 50 varicose-vein-related public queries from Google Trends and Baidu Zhidao over the past 5 years. Baidu Zhidao queries were translated into English for comparison, and benchmark questions were classified as showing direct alignment, thematic alignment, or no clear alignment. The full raw query lists and benchmark-to-query crosswalk are provided in Supplementary Tables S1C–E. Each question was mapped a priori to the most relevant Chinese and/or international guideline module(s), as appropriate. Mapping discrepancies were resolved through consensus and documented with anchor PMIDs in Supplementary Tables S1A, B (2, 4, 5, 21–23).

### Models and query procedure

Five LLMs were evaluated through their official web interfaces under default settings: ChatGPT-5.2 (release date: December 11, 2025), DeepSeek-V3.2 (release date: December 1, 2025), Gemini 3 Pro (release date: November 18, 2025), Grok 4.1 (release date: November 17, 2025), and Qwen3-Max (release date: January 26, 2026). Model identifiers were documented exactly as displayed in the official web interfaces at the time of querying.

Queries were conducted within a prespecified data-collection window (February 10–12, 2026, Asia/Singapore time) using Google Chrome. All platforms were accessed through standard consumer user accounts. There were no APIs, fine-tuning, or external tools/plugins used beyond each product's default functionality. If optional platform features related to browsing/grounding/citations were available, they were not manually modified (left at default). There were no optional browsing/grounding/citation features enabled or disabled beyond each platform's default behavior. Any baseline differences were treated as inherent platform characteristics. Prompts were submitted verbatim without rephrasing, additional context, or follow-up messages (one-shot per prompt) to approximate novice user interactions.

To minimize conversational carryover, sessions were conducted in browser privacy mode. Each prompt was entered in a new chat. The cache was cleared between model sessions. After responses were collected and exported, the corresponding conversations were deleted from the interface. These measures were intended to limit carryover and personalization, although complete elimination of platform-level personalization cannot be ensured.

Prespecified handling of refusals and technical failures was as follows: refusals, safety-policy messages, or otherwise non-responsive outputs were retained as returned and scored accordingly (i.e., no selective regeneration). If a transient technical issue produced no output, the same prompt was resubmitted up to three times until a response was obtained. There were no outputs missing in the final dataset.

### Outcome measures

#### Informational quality and verifiability indicators

Responses were independently assessed using four validated instruments: DISCERN total score (16–80), EQIP total (% yes; 0–100), Global Quality Score (GQS) (1–5), and the Journal of the American Medical Association (JAMA) benchmark total score (0–4). For all four instruments, higher scores indicate superior performance. These instruments assess patient-oriented informational quality, presentation, and transparency-related features, but they do not directly determine claim-level clinical accuracy, potential harm, safety, or formal guideline concordance. Because the JAMA benchmark was originally developed for health information websites, we applied it here pragmatically as a structured proxy for visible attribution and verifiability-related transparency in LLM-generated responses, rather than as a validated measure of broader transparency for conversational artificial intelligence (AI) outputs. Accordingly, some JAMA domains—particularly authorship, attribution, disclosure, and currency—may not map perfectly onto one-shot chatbot responses delivered through consumer interfaces. Therefore, higher scores should be interpreted as indicating better performance on these measured constructs rather than confirming that the content was medically correct, guideline-concordant, or safe. Operational definitions and item-level scoring criteria for each instrument are detailed in Supplementary Methods. No formal adjudication of factual correctness, guideline concordance, potential for harm, or clinical safety was performed in the present study.

### Blinding and rating workflow

Model outputs were exported immediately after generation and compiled into a standardized, anonymized rating workbook. Branding and interface metadata were removed. Each response was assigned a random alphanumeric ID, and response order was randomized within question blocks. Two independent raters (clinicians with >10 years of clinical experience) assessed responses offline and were provided only the question prompt and the corresponding response text. Interrater agreement was quantified before discrepancy resolution (Table 2). Rating discrepancies were addressed through consensus/adjudication according to a prespecified procedure (Table 2).

**Table 2:** Interrater agreement for primary outcomes (N = 100 model–question units).

Outcome (scale)	Agreement statistic	Value
DISCERN total (16–80)	ICC(A,1), 2-way random, absolute agreement	0.913
EQIP total (% yes; 0–100)	ICC(A,1), 2-way random, absolute agreement	0.859
GQS (1–5)	Weighted Cohen's κ (quadratic)	0.883
JAMA total (0–4)	Weighted Cohen's κ (quadratic)	0.864

Interrater agreement was assessed across 100 model–question units (5 models × 20 questions). For continuous total scores (DISCERN total and EQIP total), ICC(A,1) (two-way random-effects, absolute agreement) was used. For ordinal ratings (GQS and JAMA total scores), weighted Cohen's κ (quadratic weights) was applied. Ratings were conducted independently, and agreement statistics were calculated prior to resolving any discrepancies. EQIP, Ensuring Quality Information for Patients; GQS, Global Quality Score; ICC(A,1), intraclass correlation coefficient (two-way random-effects, absolute agreement, single measurement); JAMA, Journal of the American Medical Association.

### Readability

Readability was calculated at the response level using six indices: Automated Readability Index (ARI), Flesch Reading Ease Score (FRES), Gunning Fog Index (GFI), Flesch–Kincaid Grade Level (FKGL), Coleman–Liau Index (CL), and Simple Measure of Gobbledygook (SMOG). Because individual readability formulas emphasize different textual features and are correlated but not interchangeable, we used a panel of commonly reported indices to provide a broader characterization of reading burden rather than relying on a single formula. Responses were analyzed in their generated form (plain-text copy), with a uniform preprocessing rule applied across all responses (removal of non-content interface artifacts only; URLs and bullet formatting retained as text if present). Scores were generated using the same online calculator and identical settings for all responses (readabilityformulas.com, accessed February 12, 2026). Each response was processed three times to verify computational stability. Identical outputs were documented. Because readability calculators may vary in preprocessing and implementation specifications, absolute values should be interpreted as tool-dependent. However, all models were processed under identical conditions, supporting valid relative comparisons. For interpretability, lower values indicate greater readability for ARI/GFI/FKGL/CL/SMOG, whereas higher values indicate greater readability for FRES.

### Statistical analysis

Primary outcomes comprised the four informational quality and transparency-proxy instruments (DISCERN, EQIP, GQS, and JAMA benchmark totals). Readability indices were considered secondary outcomes. Response length was additionally quantified as word count per response and summarized descriptively by model [mean ± SD; median (Q1, Q3)] to aid interpretation of readability findings (Supplementary Table S3). Analyses were conducted using IBM SPSS Statistics 29.0 and R (version 4.3.2). Descriptive statistics are presented as mean ± SD and median [Q1, Q3] for each model (*n* = 20 responses/model) (Tables 3, 4). Overall between-model differences were evaluated using the Friedman test (within-question paired; *n* = 20; *k* = 5), with Kendall's W reported as the effect size (Table 5). In interpretation, adjusted *P-values* were considered alongside effect-size magnitude, because small effect sizes and clustered score distributions may limit the practical meaning of nominal between-model differences. This approach was prespecified because each question generated matched responses across all five models, creating a within-question paired design, and because several outcomes were ordinal or bounded and not assumed to be normally distributed. Accordingly, the Friedman test provided an assumption-light global comparison, while Kendall's W quantified the magnitude of between-model separation. Holm adjustment was applied across the 10 outcomes in Table 5. A sensitivity Holm adjustment restricted to the four primary outcomes is reported in the Results and produced consistent inferences. Alternative approaches such as mixed-effects models may be useful in future studies to account more flexibly for question-level heterogeneity, but were not used here because the present benchmark was designed around a small matched question set and a transparent non-parametric paired-comparison framework.

**Table 3:** Reliability scores across models (*n* = 20 questions per model).

Model	DISCERN total score (16–80)	EQIP total (% yes; 0–100)	JAMA total score (0–4)	GQS (1–5)
ChatGPT 5.2	48.85 ± 8.05 51.00 [43.25, 54.00]	71.50 ± 5.40 70.00 [68.75, 75.00]	0.10 ± 0.31 0.00 [0.00, 0.00]	3.90 ± 0.55 4.00 [4.00, 4.00]
DeepSeek-V3.2	50.75 ± 6.34 52.00 [47.00, 55.25]	74.25 ± 4.94 75.00 [70.00, 80.00]	0.00 ± 0.00 0.00 [0.00, 0.00]	4.30 ± 0.47 4.00 [4.00, 5.00]
Gemini 3 Pro	47.55 ± 6.46 49.00 [44.25, 51.50]	71.75 ± 6.34 70.00 [65.00, 76.25]	0.00 ± 0.00 0.00 [0.00, 0.00]	4.05 ± 0.51 4.00 [4.00, 4.00]
Grok 4.1	49.10 ± 5.59 50.00 [46.75, 54.00]	72.75 ± 6.38 70.00 [70.00, 75.00]	0.25 ± 0.44 0.00 [0.00, 0.25]	4.05 ± 0.39 4.00 [4.00, 4.00]
Qwen3-Max	46.50 ± 6.95 48.00 [40.75, 52.25]	71.75 ± 6.34 70.00 [68.75, 75.00]	0.10 ± 0.31 0.00 [0.00, 0.00]	3.90 ± 0.31 4.00 [4.00, 4.00]

Cells display the mean ± SD on the first line and the median [Q1, Q3] on the second line (n = 20 responses per model). Higher scores indicate better informational quality/transparency-proxy performance. DISCERN range: 16–80; EQIP: 0–100; JAMA: 0–4; GQS: 1–5. EQIP, Ensuring Quality Information for Patients; GQS, Global Quality Score; JAMA, Journal of the American Medical Association; Q1, first quartile; Q3, third quartile; SD, standard deviation.

**Table 4:** Readability metrics across models (*n* = 20 questions per model).

Model	ARI	FRES	GFI	FKGL	CL	SMOG
ChatGPT 5.2	12.41 ± 2.48 12.75 [10.75, 13.06]	49.30 ± 13.08 49.50 [41.50, 57.25]	11.45 ± 1.89 11.55 [9.88, 13.22]	10.01 ± 2.29 10.14 [8.49, 11.32]	13.51 ± 2.02 13.05 [11.92, 14.81]	9.44 ± 1.61 9.46 [8.32, 10.51]
DeepSeek-V3.2	12.42 ± 1.37 12.18 [11.25, 13.36]	45.95 ± 5.95 46.50 [42.75, 50.00]	12.35 ± 1.13 12.50 [11.73, 12.93]	10.61 ± 1.05 10.43 [9.88, 11.20]	13.64 ± 1.28 13.54 [12.59, 14.57]	10.16 ± 0.87 10.17 [9.61, 10.61]
Gemini 3 Pro	11.98 ± 1.72 12.38 [11.03, 13.26]	50.55 ± 7.22 49.00 [45.75, 54.00]	11.76 ± 1.45 11.95 [11.25, 12.75]	10.41 ± 1.45 10.89 [9.80, 11.54]	12.53 ± 1.25 12.54 [11.81, 13.47]	9.85 ± 1.20 10.10 [9.29, 10.71]
Grok 4.1	14.60 ± 2.32 14.82 [13.21, 16.20]	37.30 ± 11.10 36.00 [29.00, 47.00]	12.94 ± 2.09 13.85 [11.38, 14.43]	12.20 ± 2.07 12.59 [10.99, 13.53]	15.49 ± 1.80 15.63 [14.01, 16.63]	10.93 ± 1.88 11.12 [9.56, 12.45]
Qwen3-Max	14.20 ± 1.89 14.38 [12.58, 15.76]	29.95 ± 9.00 30.50 [25.75, 35.50]	14.68 ± 1.80 14.80 [13.50, 15.78]	12.42 ± 1.56 12.21 [11.37, 13.81]	16.82 ± 1.66 16.48 [15.46, 17.90]	11.00 ± 1.40 11.23 [9.96, 11.91]

Cells display the mean ± SD on the first line and the median [Q1, Q3] on the second line (n = 20 responses per model). For ARI, GFI, FKGL, CL, and SMOG, lower values indicate easier readability; for FRES, higher values indicate easier readability. Lower values indicate easier readability for ARI/GFI/FKGL/CL/SMOG; higher values indicate easier readability for FRES. ARI, Automated Readability Index; CL, Coleman–Liau Index; FKGL, Flesch–Kincaid Grade Level; FRES, Flesch Reading Ease Score; GFI, Gunning Fog Index; Q1, first quartile; Q3, third quartile; SD, standard deviation; SMOG, Simple Measure of Gobbledygook.

**Table 5:** Overall between-model differences (Friedman test; within-question paired, *n* = 20; *k* = 5).

Outcome	Friedman *χ2*	*df*	*P*	Kendall W	*P* (Holm, across outcomes)
DISCERN total score (16–80)	11.266	4	0.024	0.141	0.071
EQIP total (% yes; 0–100)	5.273	4	0.260	0.066	0.260
JAMA total score (0–4)	12.000	4	0.017	0.150	0.069
GQS (1–5)	8.315	4	0.081	0.104	0.161
ARI	26.360	4	< 0.001	0.330	< 0.001
FRES	54.020	4	< 0.001	0.675	< 0.001
GFI	39.709	4	< 0.001	0.496	< 0.001
FKGL	28.760	4	< 0.001	0.359	< 0.001
CL	62.520	4	< 0.001	0.781	< 0.001
SMOG	16.760	4	0.002	0.209	0.011

Holm adjustment in the last column is applied across the 10 outcomes in this table. Overall between-model differences were assessed using the Friedman test (within-question paired; n = 20; k = 5), with Kendall's W reported as an effect size. The Holm-adjusted P-value in the last column is adjusted across the 10 outcomes in this table. ARI, Automated Readability Index; CL, Coleman–Liau Index; df, degrees of freedom; EQIP, Ensuring Quality Information for Patients; FKGL, Flesch–Kincaid Grade Level; FRES, Flesch Reading Ease Score; GFI, Gunning Fog Index; GQS, Global Quality Score; JAMA, Journal of the American Medical Association; SMOG, Simple Measure of Gobbledygook.

For pairwise comparisons, two-sided Wilcoxon signed-rank tests were conducted for each outcome across all 10 model pairs, paired by question (*n* = 20). Holm adjustment was applied within each outcome across the 10 pairwise comparisons (Supplementary Table S2). Raw (unadjusted) *P-values* for the primary outcomes are provided in Table 6 for descriptive completeness only and should be interpreted as exploratory rather than confirmatory, particularly where overall adjusted analyses did not support statistically robust between-model differences.

**Table 6:** Pairwise comparison raw *P-values* (Wilcoxon signed-rank test; two-sided).

Comparison	DISCERN	GQS	EQIP	JAMA
ChatGPT 5.2 vs. DeepSeek-V3.2	0.1905	0.0456	0.0532	0.1573
ChatGPT 5.2 vs. Gemini 3 Pro	0.2748	0.2568	0.9043	0.1573
ChatGPT 5.2 vs. Grok 4.1	0.9132	0.3657	0.4462	0.0833
ChatGPT 5.2 vs. Qwen3-Max	0.1017	1	0.8709	1
DeepSeek-V3.2 vs. Gemini 3 Pro	0.0154	0.1597	0.1896	1
DeepSeek-V3.2 vs. Grok 4.1	0.2086	0.0588	0.2334	0.0253
DeepSeek-V3.2 vs. Qwen3-Max	0.0107	0.0114	0.0957	0.1573
Gemini 3 Pro vs. Grok 4.1	0.2037	1	0.6537	0.0253
Gemini 3 Pro vs. Qwen3-Max	0.3525	0.2568	0.9046	0.1573
Grok 4.1 vs. Qwen3-Max	0.0726	0.1797	0.4710	0.1797

Values are raw (unadjusted) two-sided P-values from within-question paired Wilcoxon signed-rank tests (n = 20 pairs per comparison). Holm-adjusted P-values (within each outcome across the 10 pairwise comparisons) are provided in Supplementary Table S2; Figure 5 visualizes these adjusted values as –log10(p_holm). EQIP, Ensuring Quality Information for Patients; GQS, Global Quality Score; JAMA, Journal of the American Medical Association.

### Data normalization and figure computations

**Figure 1:**
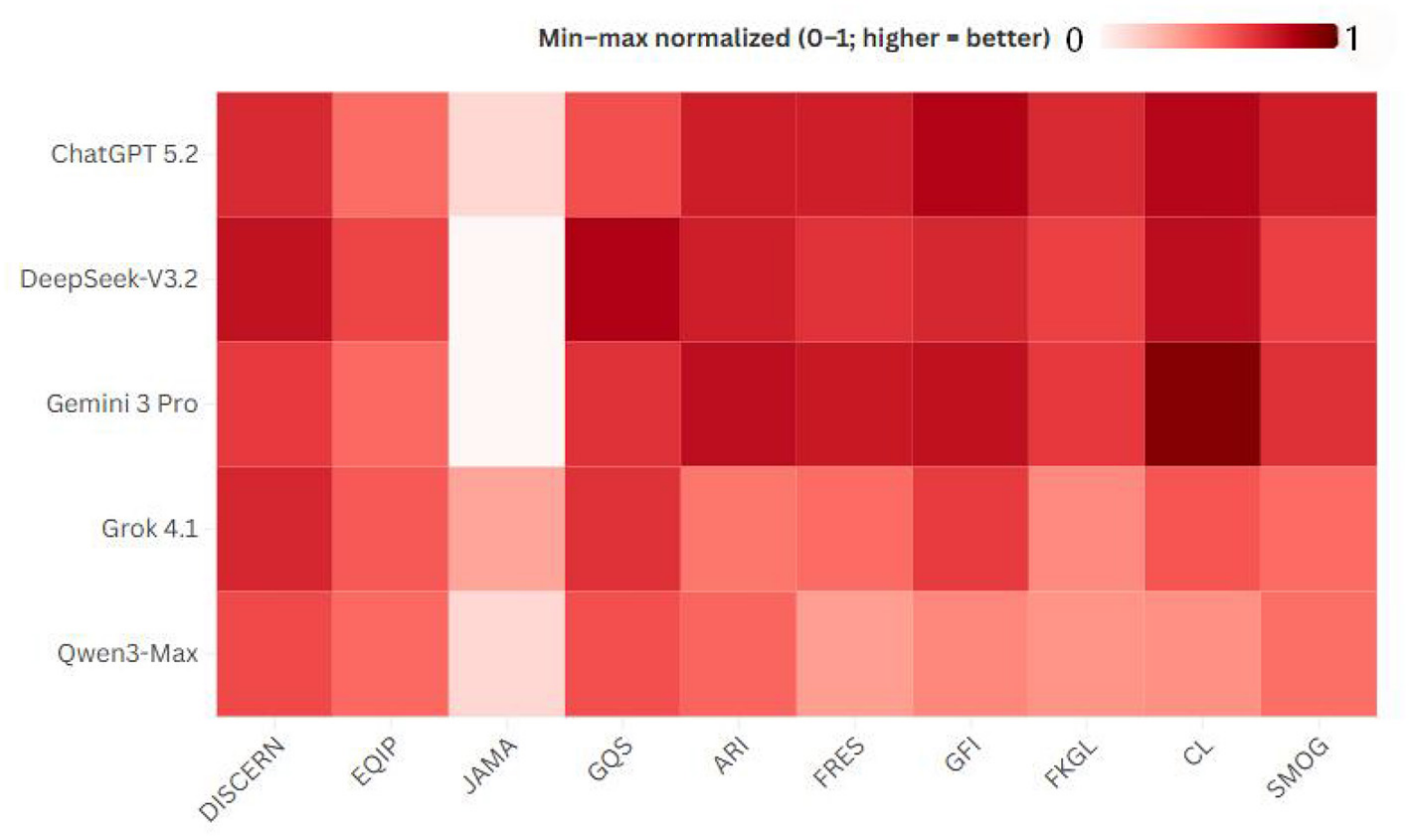
Min–max normalized heatmap of reliability and readability metrics across 5 LLMs (*n* = 20 responses per model). Values were min–max scaled to 0–1 within each metric across all 100 responses (higher = better); darker shading denotes superior normalized performance. For readability indices in which lower values correspond to easier readability (ARI/GFI/FKGL/CL/SMOG), directionality was aligned such that higher normalized values reflect easier readability; FRES retained its original direction (higher = easier). Model-level cells represent the mean of response-level normalized values. Therefore, values may not reach 0 or 1 despite normalization on a 0–1 scale. These heatmap values represent descriptive min–max normalized scores rather than effect sizes and were used for visualization only.

**Figure 2:**
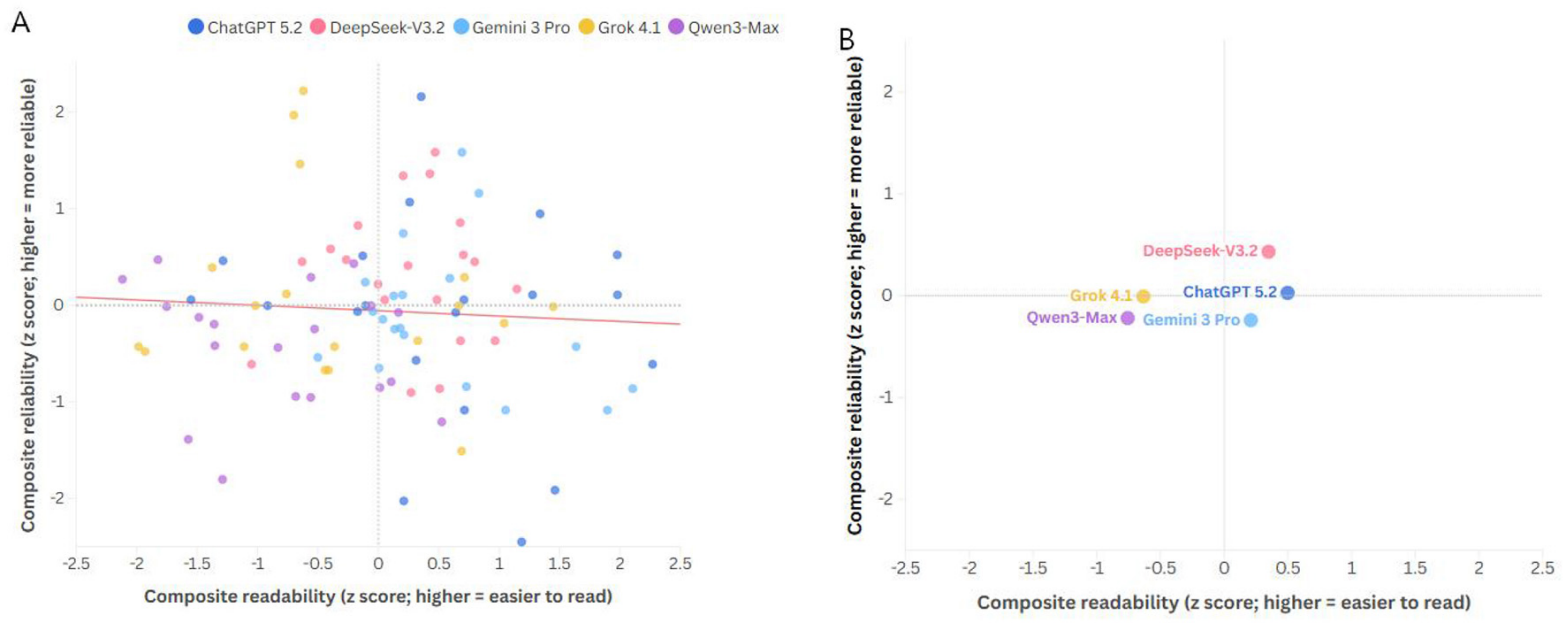
Trade-off between composite readability and composite reliability across models. **(A)** Response-level trade-off (*n* = 100). **(B)** Model-level medians (*n* = 20 per model). Composite scores were calculated at the response level using 0–1 min–max scaling across all 100 responses. The reliability composite is the unweighted mean of scaled DISCERN total, EQIP total, JAMA total, and GQS (higher values indicate better quality/verifiability indicators). The readability composite is the unweighted mean of direction-aligned readability indices: aligned = scaled for FRES and aligned = 1 – scaled for ARI/GFI/FKGL/CL/SMOG (higher values indicate easier readability). The solid trend line in panel A represents the least-squares linear fit across all response-level points, summarizing the overall association between composite readability and composite reliability. Because the 100 response-level observations are nested within 20 matched questions, panel A is descriptive and should not be interpreted as showing 100 statistically independent observations.

To ensure consistent directionality in visualization, response-level 0–1 min–max scaling was applied within each metric across all 100 responses, yielding the scaled value: s = (x – min) / (max – min). Subsequently, direction alignment was implemented for readability metrics so that higher values correspond to easier readability: for FRES, the aligned value = s; for ARI/GFI/FKGL/CL/SMOG, the aligned value = 1 – s. These scaled values and composite scores were used exclusively for visualization and descriptive trade-off plots (Figures 1, 2) and were not incorporated into statistical inference. Because min–max scaling may be influenced by extreme values, the results should be interpreted as descriptive normalization.

**Figure 3:**
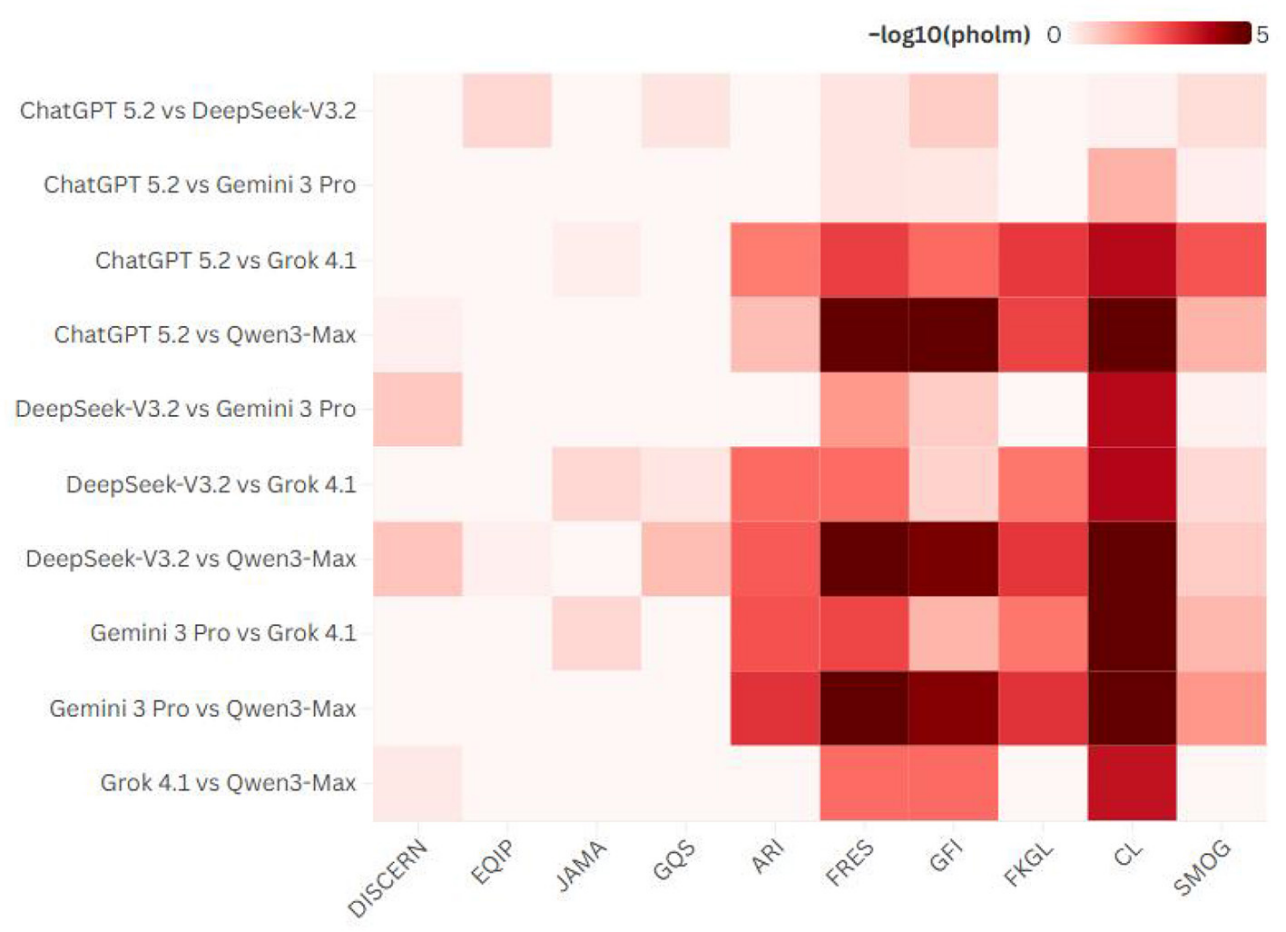
Pairwise differences in quality, verifiability indicators, and readability across LLMs (Holm-adjusted). The heatmap presents pairwise model comparisons (rows) across 10 outcome metrics (columns). Each cell reports –log10(p_holm), where p_holm denotes the Holm-adjusted *P-value* derived from the corresponding two-sided within-question paired Wilcoxon signed-rank test (*n* = 20 pairs per comparison); larger values indicate stronger evidence of a between-model difference. Holm adjustment was applied within each outcome across the 10 pairwise comparisons. Raw (unadjusted) *P-values* for the primary outcomes are provided in Table 6.

For Figure 1, model-level heatmap cells represent the mean aligned scaled value across the 20 responses generated by each model. For Figure 2, composite scores were calculated at the response level as unweighted means of aligned scaled metrics: reliability composite = mean (aligned DISCERN, EQIP, JAMA, GQS); readability composite = mean (aligned ARI, FRES, GFI, FKGL, CL, SMOG). Model-level composite values were then summarized using medians across each model's 20 responses. For Figure 3, heatmap cells display –log10(p_holm), where p_holm denotes the Holm-adjusted *P*-*value* for the corresponding pairwise comparison within each outcome (Supplementary Table S2). The normalized values shown in Figure 1 are descriptive visualization values and should not be interpreted as effect sizes. In addition, because panel A of Figure 2 displays 100 response-level observations derived from 20 matched questions (five model-specific responses per question), these points are clustered within questions and should not be interpreted as statistically independent observations. Accordingly, the response-level scatter and fitted line are presented for descriptive visualization only and were not used for inferential testing.

### Reporting guidelines and ethics

This study was reported in accordance with the CHART statement (16). The analysis assessed outputs generated by publicly accessible AI systems using prompts derived from established guidelines. There were no human participants involved, and no identifiable private data was collected or analyzed.

## Results

### Patient-facing question set and analytic units

The final benchmark comprised 20 single-intent, patient-facing VV questions spanning the care pathway (Table 1). Five publicly available LLMs (ChatGPT 5.2, DeepSeek-V3.2, Gemini 3 Pro, Grok 4.1, and Qwen3-Max) were queried using the identical question set, resulting in 100 model–question responses (5 models × 20 questions). Each model–question response constituted the analytic unit for all evaluated outcomes.

### Interrater agreement

Interrater agreement across the 100 model–question units was high for all prespecified primary outcomes (Table 2). Agreement was excellent for DISCERN total [ICC(A,1) = 0.913] and EQIP total [ICC(A,1) = 0.859], and remained comparably high for ordinal outcomes assessed with quadratic-weighted Cohen's κ (GQS κ = 0.883; JAMA total κ = 0.864).

### Informational quality and transparency-proxy performance

**Figure 4:**
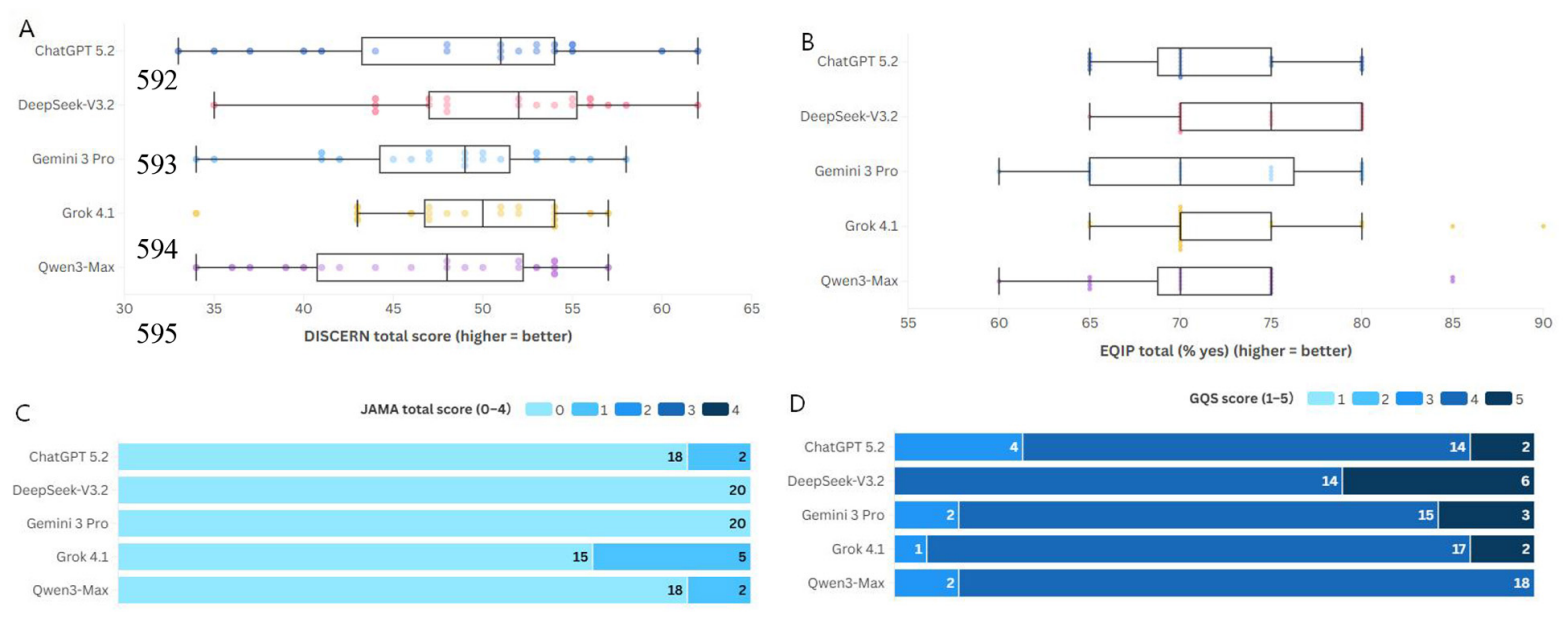
Distribution of reliability and transparency-proxy scores across 5 LLMs in response to 20 VV questions (*n* = 100 responses; 20 per model). **(A)** DISCERN total (16–80). **(B)** EQIP total (% yes; 0–100). **(C)** JAMA benchmark total (0–4). **(D)** GQS (1–5). Higher scores denote better informational quality or stronger verifiability indicators. Boxes represent the interquartile range (IQR) with the median shown as a horizontal line; whiskers extend to 1.5 × IQR; points correspond to individual question-level outputs (one point per response).

Model-level outcomes are summarized in Table 3, with response-level distributions presented in Figure 4. DISCERN totals demonstrated modest between-model differentiation, ranging from 46.50 ± 6.95 [median: 48.00 (40.75, 52.25)] for Qwen3-Max to 50.75 ± 6.34 [median: 52.00 (47.00, 55.25)] for DeepSeek-V3.2. ChatGPT 5.2 achieved 48.85 ± 8.05 [median: 51.00 (43.25, 54.00)] (Table 3). EQIP totals were relatively clustered (mean range: 71.50–74.25), with DeepSeek-V3.2 attaining the highest value at 74.25 ± 4.94 [median: 75.00 (70.00, 80.00)], whereas most other models were centered at a median of 70.00 (Table 3). GQS ratings remained consistently high across models (means: 3.90–4.30), with medians of 4.00 for all models (Table 3; Figure 4). In contrast, JAMA benchmark totals approximated zero across models (means: 0.00–0.25), with medians of 0.00. Grok 4.1 exhibited the highest mean JAMA total [0.25 ± 0.44; median: 0.00 (0.00, 0.25)] (Table 3; Figure 4).

### Readability performance

**Figure 5:**
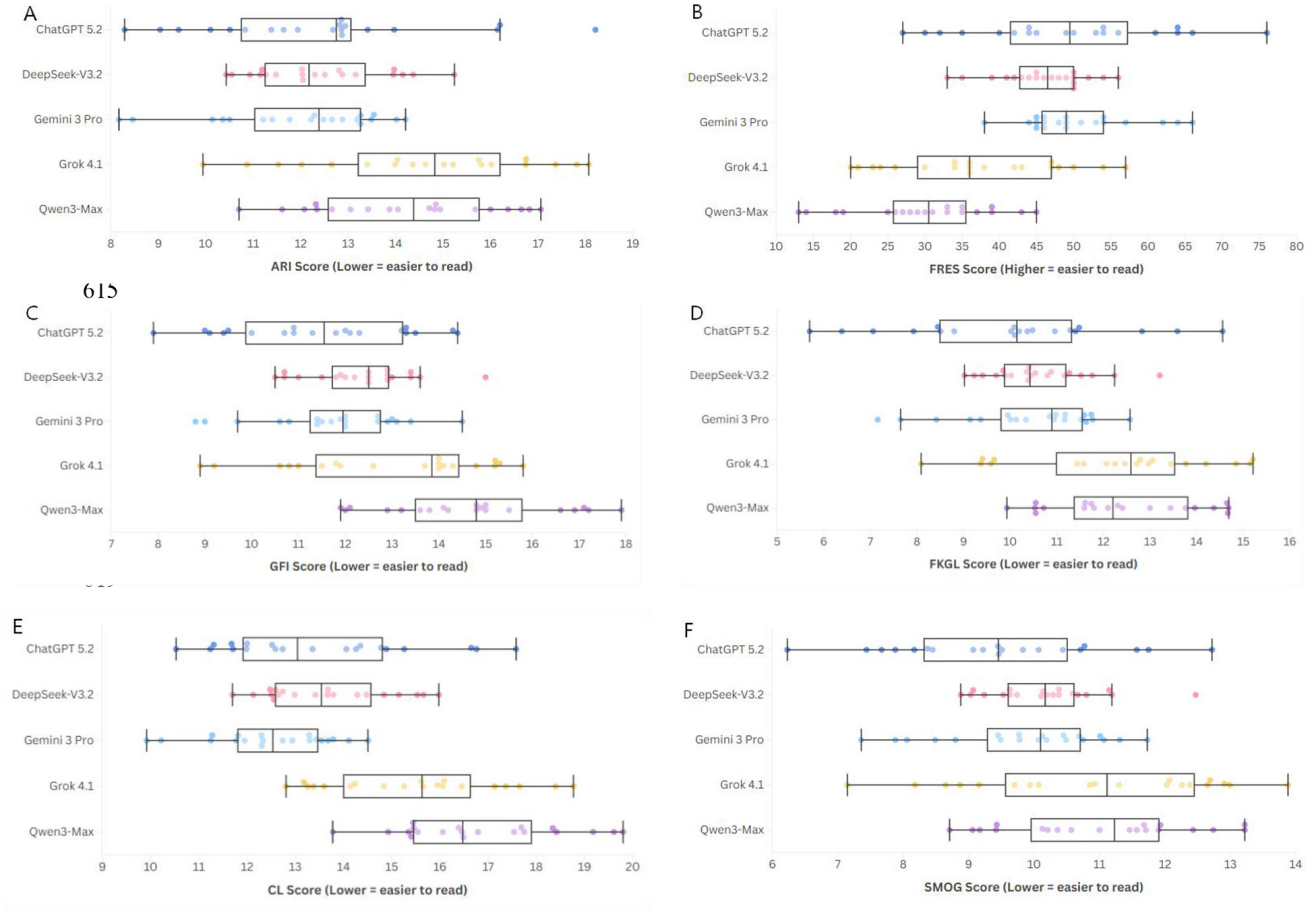
Distribution of readability scores across 5 LLMs in response to 20 VV questions (*n* = 100 responses; 20 per model). Box-and-whisker plots with overlaid individual data points compare readability across six indices: **(A)** ARI, **(B)** FRES, **(C)** GFI, **(D)** FKGL, **(E)** CL, and **(F)** SMOG. Lower values indicate easier readability for ARI/GFI/FKGL/CL/SMOG, whereas higher values indicate easier readability for FRES. Reference thresholds for sixth-grade readability are displayed (scores < 6 for ARI/GFI/FKGL/CL/SMOG and FRES ≥ 80). Boxes represent the interquartile range (IQR) with the median indicated by a horizontal line; whiskers extend to 1.5 × IQR; points correspond to individual question-level outputs (one point per response).

Readability outcomes are presented in Table 4 and illustrated in Figure 5. Across grade-level indices (ARI/GFI/FKGL/CL/SMOG; lower values indicate easier readability), Gemini 3 Pro consistently demonstrated lower scores, whereas Grok 4.1 and Qwen3-Max produced comparatively higher values. For example, ARI ranged from 11.98 ± 1.72 [median: 12.38 (11.03, 13.26)] for Gemini 3 Pro to 14.60 ± 2.32 [median: 14.82 (13.21, 16.20)] for Grok 4.1 (Table 4). CL ranged from 12.53 ± 1.25 [median: 12.54 (11.81, 13.47)] for Gemini 3 Pro to 16.82 ± 1.66 [median: 16.48 (15.46, 17.90)] for Qwen3-Max (Table 4). FRES (higher values indicate easier readability) was highest for Gemini 3 Pro [50.55 ± 7.22; median: 49.00 (45.75, 54.00)] and ChatGPT 5.2 [49.30 ± 13.08; median: 49.50 (41.50, 57.25)], intermediate for DeepSeek-V3.2 [45.95 ± 5.95; median: 46.50 (42.75, 50.00)], and lowest for Qwen3-Max [29.95 ± 9.00; median: 30.50 (25.75, 35.50)] (Table 4; Figure 5). Based on the sixth-grade reference thresholds shown in Figure 5, all models exceeded grade-level cutoffs for ARI/GFI/FKGL/CL/SMOG and exhibited FRES values < 80.

### Overall between-model differences and normalized patterns

Overall within-question paired comparisons are presented in Table 5. For primary outcomes, between-model differences were small (Kendall's *W* = 0.066–0.150). DISCERN [Friedman χ^2^(4) = 11.266, *P* = 0.024; W = 0.141] and JAMA [χ^2^(4) = 12.000, *P* = 0.017; W = 0.150] demonstrated nominal overall differences across models. However, neither remained < 0.05 following Holm adjustment across the 10 outcomes (Holm *P* = 0.071 and 0.069, respectively). Given the modest benchmark size (20 matched questions), these null findings after multiplicity correction should be interpreted cautiously, as the study may have had limited power to detect small between-model differences in the primary outcomes. EQIP [χ^2^(4) = 5.273, *P* = 0.260; W = 0.066] and GQS [χ^2^(4) = 8.315, *P* = 0.081; W = 0.104] showed no overall between-model differences (Table 5). A sensitivity Holm adjustment restricted to the four primary outcomes similarly did not produce adjusted *P-values* < 0.05 for DISCERN or JAMA (adjusted *P* = 0.072 and 0.068, respectively).

In contrast, all readability indices differed significantly across models (ARI/FRES/GFI/FKGL/CL: all *P* < 0.001; SMOG *P* = 0.002), with moderate-to-large effect sizes and Holm-adjusted statistical significance retained for each readability outcome (Holm *P* < 0.001 for ARI/FRES/GFI/FKGL/CL; Holm *P* = 0.011 for SMOG). The largest effect sizes were observed for CL (*W* = 0.781) and FRES (*W* = 0.675), followed by GFI (*W* = 0.496) (Table 5). Min–max normalized model profiles across outcomes are summarized in Figure 1. Composite readability–reliability associations at both the response level and model-median level are presented in Figure 2. However, the response-level panel should be interpreted descriptively because the plotted observations are clustered within the same set of 20 matched questions and are therefore not fully independent.

### Pairwise comparisons

Holm-adjusted pairwise results across all 10 outcomes are summarized in Figure 3 [–log10(p_holm), with Holm adjustment applied within each outcome across the 10 pairwise model comparisons]. Raw (unadjusted) pairwise *P-values* for the primary outcomes are presented in Table 6. For DISCERN, nominal pairwise differences were identified between DeepSeek-V3.2 and Gemini 3 Pro (*P* = 0.0154), as well as between DeepSeek-V3.2 and Qwen3-Max (*P* = 0.0107). For GQS, nominal differences were observed between ChatGPT 5.2 and DeepSeek-V3.2 (*P* = 0.0456) and between DeepSeek-V3.2 and Qwen3-Max (*P* = 0.0114). For JAMA, nominal differences were detected between DeepSeek-V3.2 and Grok 4.1 (*P* = 0.0253) and between Gemini 3 Pro and Grok 4.1 (*P* = 0.0253), whereas EQIP demonstrated no nominally significant pairwise differences (Table 6).

## Discussion

In this benchmarking study, the informational quality, verifiability indicators, and readability of five publicly available LLMs were compared across 20 patient-facing VV questions. Interrater reliability was high for all prespecified outcomes (Table 2), supporting the robustness of subsequent between-model comparisons. Across models, patient-oriented quality metrics showed only modest differences, whereas performance on verifiability indicators was uniformly low, particularly for the JAMA benchmark, despite generally favorable global ratings (Table 3; Figure 4). In contrast, readability varied substantially across models on all indices (Table 4; Figure 5), with normalized profiles and composite trade-offs illustrated in Figures 1 and 2 and pairwise results summarized in Figure 3 (with primary-outcome raw *P-values* provided in Table 6). Beyond reporting model-level differences, this study contributes a disease-specific, guideline-anchored, and deployment-oriented benchmark that helps foreground auditability as a practical evaluative consideration for patient-facing LLM outputs in digital public health.

Reliability of scoring and interpretability of comparisons. Agreement was excellent for DISCERN and EQIP total scores and remained comparably high for ordinal outcomes (Table 2), indicating that the observed inter-model patterns are unlikely to be attributable to rater inconsistency. This is particularly important because assessments of LLM outputs have been criticized for methodological heterogeneity and incomplete reporting, which may compromise reproducibility and limit cross-study comparability (17, 24, 25). Therefore, these findings establish a robust foundation for interpreting model-level differences and identifying actionable failure modes.

Informational quality and verifiability indicators: a polished but poorly auditable pattern. However, because the JAMA benchmark was originally developed for health information websites rather than conversational AI outputs, its application in this context should be interpreted cautiously, and low scores may reflect both limited visible verifiability information and imperfect instrument–context fit. DISCERN and EQIP total scores clustered within a relatively narrow range across models (Table 3; Figure 4), indicating broadly comparable performance on patient-oriented quality criteria within this VV domain, and GQS medians were uniformly 4.0 (Table 3). In contrast, JAMA benchmark totals approximated zero for all models (Table 3; Figure 4), indicating that key provenance and currency components (e.g., authorship/attribution, sourcing, disclosure, and update information) were largely absent. Notably, the JAMA benchmark functions as a proxy for structural attribution and visible verifiability, rather than a measure of factual accuracy or broader transparency in a comprehensive sense. Accordingly, low scores primarily suggest that users are not provided with sufficient attribution, sourcing, disclosure, or currency cues to verify claims, contextualize uncertainty, or differentiate guideline-concordant recommendations from plausible-sounding inaccuracies (26, 27). However, because the JAMA benchmark was originally developed for health information websites rather than conversational AI outputs, its application in this context should be interpreted cautiously. The uniformly low scores observed here should therefore not be understood as a direct measure of deficient transparency overall, but rather as indicating limited visible attribution, sourcing, disclosure, and currency cues, together with imperfect instrument–context fit. The centrality of verifiability has long been emphasized in frameworks evaluating the quality of medical information on the internet, and this consideration assumes greater significance in the context of LLM-generated content given documented risks of fabricated or erroneous references and the potential for citation-like material to convey unwarranted credibility (18, 28, 29).

Readability: large between-model differences, but none meet common accessibility targets. Readability outcomes exhibited the strongest between-model differentiation, with large effect sizes across several indices (Table 5). However, all models exceeded widely cited sixth-grade benchmarks on grade-level measures, and FRES values remained substantially below the frequently referenced accessibility threshold of ≥80 (Table 4; Figure 5). This pattern should not be interpreted as indicating that all observed complexity was avoidable, because some reading burden is likely inherent to patient-facing explanations of VV and chronic venous disease, particularly when discussing duplex ultrasound evaluation, compression therapy, procedural options, recurrence prevention, and warning symptoms (9, 10). Nonetheless, the pronounced between-model differences observed here suggest that model choice and generation style can meaningfully alter the reading burden. Not all complexity is equivalent. Some reflects necessary clinical content, whereas some may arise from avoidable linguistic features such as excessive verbosity, long sentences, unexplained jargon, and limited plain-language organization. Response-length statistics are provided in Supplementary Table S3. Mean word count varied substantially across models, from 268.60 ± 68.62 words for Grok 4.1 to 554.75 ± 83.73 words for DeepSeek-V3.2, suggesting that between-model differences in verbosity may have contributed in part to the observed readability differences. Because readability scores may be affected by response length and formatting, we additionally reported response-length statistics to improve interpretability. These data suggest that between-model differences in verbosity may have partially contributed to the observed readability separation. Future benchmarks should consider length-adjusted readability metrics or constrained-generation prompts.

Implications for auditability, interpretability, and evaluation standards. These findings also have practical implications for clinicians, patients, and evaluation frameworks. For clinicians, publicly accessible LLM outputs may serve as adjunctive informational tools, but should not be treated as substitutes for clinical assessment, particularly when responses do not provide verifiable sources, explicit uncertainty statements, or clear information boundaries. For patients, fluent responses may create a misleading impression of reliability when provenance, currency, and limitations are not clearly stated, while readability barriers may further affect comprehension. In practice, the key issue is not complexity *per se*, but whether necessary medical information is communicated in a form that patients can readily understand, interpret, and act upon. Excessive avoidable complexity may reduce comprehension of core management options, obscure the significance of red-flag symptoms, and disproportionately disadvantage readers with limited health literacy. From an implementation and evaluation perspective, future assessment approaches for public-facing medical AI should extend beyond structural attribution alone. They should also consider provenance reporting, verifiable citation practices, uncertainty communication, information currency, and readability for intended users. Importantly, auditability and verifiability should be interpreted as complementary evaluation dimensions rather than substitutes for formal assessment of factual accuracy, guideline concordance, and clinical safety. The observed pattern supports a pragmatic design objective: improving auditability by design in patient-facing LLM outputs. Patient-facing responses should explicitly provide (1) the evidentiary basis (guideline organization/name, publication year, and, where feasible, the specific recommendation module), (2) response currency (date of generation and guideline currency), (3) explicit uncertainty statements and contraindication prompts, and (4) clearly defined escalation triggers for urgent symptoms. Such transparency is consistent with emerging reporting and evaluation standards for chatbot-delivered health advice studies (e.g., CHART) (16). Concurrently, the citation-hallucination literature supports avoiding decorative citations and instead prioritizing verifiable references that can be independently validated, with explicit labeling when no source is available for a given claim (9, 10). Given the growing public reliance on LLMs for health information, these measures are directly associated with improved trust calibration and risk mitigation (30, 31).

### Strengths and limitations

Key strengths include a standardized, single-intent question set spanning the VV care pathway, evaluation of multiple publicly available models under standardized one-shot querying, and within-question paired comparisons that reduce confounding by question content. Several limitations warrant consideration. First, this benchmark reflects a time-bound snapshot of rapidly evolving models. Outputs and safeguards may change over time. Second, although DISCERN, EQIP, GQS, and the JAMA benchmark are useful for evaluating patient-oriented informational quality, structure, and verifiability indicators, they do not directly adjudicate claim-level factual accuracy, potential for harm, clinical safety, or formal guideline concordance against a clinical gold standard. Accordingly, the present findings should not be interpreted as evidence of clinical accuracy or safety, nor as supporting comparative claims regarding the safe deployment of these models in patient care. In addition, the JAMA benchmark was originally designed for health information websites, and its use for one-shot LLM responses may therefore involve imperfect conceptual fit. Future evaluations should therefore complement such structural attribution measures with LLM-specific evaluation approaches that assess citation verifiability, uncertainty communication, provenance reporting, and other model-relevant disclosure behaviors. The present study should be understood as a pragmatic step in that direction rather than as a definitive framework. Accordingly, higher scores on these instruments do not necessarily indicate that a response is medically correct, guideline-concordant, or safe for patient decision-making; rather, these measures should be interpreted as complementary to, not substitutes for, formal evaluation of factual accuracy, guideline concordance, and clinical safety. Future work should complement these measures with formal accuracy adjudication, guideline-concordance assessment, and safety-critical error taxonomies. Third, English-only prompting enhances cross-model comparability but may not generalize to other languages or cultural contexts. Fourth, the question set was guideline derived and intentionally designed to cover decision-critical domains across the VV care pathway rather than to mirror the frequency distribution of real-world patient queries. Therefore, it may not fully capture the phrasing, prevalence, or salience of topics in routine patient information seeking. We did not formally derive questions from patient FAQs, online forums, or search logs, and no formal external Delphi-style review by experts outside the study team was performed. However, in a *post-hoc* external comparison against the top 50 varicose-vein-related public queries from Google Trends and Baidu Zhidao over the past 5 years, 5 of 20 benchmark questions showed direct alignment and an additional 7 showed thematic alignment, yielding 12 of 20 questions (60.0%) with at least partial overlap with common real-world online search intents (Supplementary Tables S1C–E). These findings support partial real-world alignment of the benchmark, but do not establish full representativeness of public patient query distributions. Future studies should consider hybrid question-set development that combines guideline-derived domains with patient-oriented sources and external expert or patient review. Fifth, the focused PubMed-only, single-query guideline search prioritized auditability but may have overlooked relevant guidance indexed under alternative terminology or published outside PubMed. Finally, readability values are partially tool-dependent and may be influenced by response length and formatting. Reproducible, script-based readability pipelines and pre-registered preprocessing rules would further strengthen comparability across studies.

An additional statistical consideration is that the benchmark included only 20 matched questions, which may have limited power to detect modest between-model differences in the primary outcomes. This is particularly relevant because the observed effect sizes for DISCERN, EQIP, GQS, and JAMA were small, and score distributions were relatively clustered across models. Accordingly, the present results do not support strong claims of model superiority for the primary informational-quality outcomes. Consequently, the loss of statistical significance after Holm correction should not be interpreted as proof of equivalence, but neither should nominal between-model differences be overstated. Rather, these findings should be understood as reflecting a combination of modest absolute differences, limited sample size, and conservative control of multiplicity. We therefore prioritize effect-size estimates and statistical uncertainty alongside adjusted *P-values*. Although alternative approaches such as mixed-effects models could provide a more flexible framework for modeling question-level heterogeneity in future studies, the present analysis used a prespecified non-parametric paired design that was well aligned with the structure of this benchmark. In addition, the response-level composite visualization in Figure 2A (*n* = 100) reflects clustered observations nested within 20 matched questions rather than 100 fully independent units. Therefore, the response-level scatter and fitted trend line should be interpreted as descriptive summaries of clustered data, whereas inferential comparisons in this study were based on the prespecified within-question paired analyses. The present benchmark should therefore be interpreted as a clinically anchored, decision-critical evaluation framework with partial real-world alignment, rather than as a prevalence-weighted reconstruction of patient search behavior.

In conclusion, across this VV benchmark, publicly available LLMs produced responses with generally favorable global quality impressions and modest differences in DISCERN/EQIP performance, but uniformly low scores on verifiability indicators and consistently suboptimal readability. These findings support prioritizing auditability, verifiable sourcing, and readability-aware generation when developing and evaluating patient-facing LLM outputs.

## Data Availability

The raw data supporting the conclusions of this article will be made available by the authors, without undue reservation.
