# Supplementary material for: Benchmarking public large language model responses to patient-facing varicose veins questions: informational quality, verifiability indicators, and readability
Source: Front Public Health. 2026 Jun 12;14:1818821. doi: 10.3389/fpubh.2026.1818821 (PMC13303600; doi:10.3389/fpubh.2026.1818821)
Supplement: Supplementary file 1 [file Table_1.docx]

***Supplementary Material***

Benchmarking Public Large Language Model Responses to Patient-Facing Varicose Veins Questions Derived from Clinical Guidelines: Informational Quality, verifiability indicators, and Readability

**1 Supplementary Methods**

**1.1 Rating instruments and scoring**

DISCERN: 16 items scored on a 5-point scale (1-5), total 16-80; higher scores indicate higher-quality consumer health information. EQIP: 20-item checklist coded Yes/No at the response level and summarized as percent Yes (Yes/20 x 100). JAMA benchmark: 4-point transparency proxy (authorship, attribution/references, disclosure, currency), summed 0-4. Global Quality Score (GQS): single-item overall quality rating (1-5).

**1.2 Readability computation**

Six readability indices were computed from each response after standardized text preprocessing: Automated Readability Index (ARI), Flesch Reading Ease Score (FRES), Gunning Fog Index (GFI), Flesch-Kincaid Grade Level (FKGL), Coleman-Liau Index (CL), and SMOG. Higher FRES indicates easier readability; higher ARI/GFI/FKGL/CL/SMOG indicate more difficult readability.

**1.3 Response length computation**
Response length was quantified as word count per response using the plain-text output after removal of non-content interface artifacts only; URLs and bullet formatting, when present, were retained as text. Word counts were summarized descriptively by model as mean ± SD and median [Q1, Q3] (Supplementary Table S3).

**1.4 Statistical analysis for Supplementary Table S2**

Two-sided Wilcoxon signed-rank tests were performed for each outcome across all 10 model pairs, paired by question (n = 20). Zero-difference pairs were handled using the Pratt method. Holm adjustment was applied within each outcome across the 10 pairwise comparisons; Supplementary Table S2 reports both raw and Holm-adjusted P values.

**1.5 Figure normalization and composites (descriptive only)**

For visualization (Figures in the main manuscript), response-level min-max scaling was applied within each metric across all 100 responses: s = (x - min)/(max - min). Readability metrics were direction-aligned so that higher values indicate easier readability (aligned = s for FRES; aligned = 1 - s for ARI/GFI/FKGL/CL/SMOG). Composite scores were unweighted means: reliability composite = mean(aligned DISCERN, EQIP, JAMA, GQS); readability composite = mean(aligned ARI, FRES, GFI, FKGL, CL, SMOG). These transformations were used only for visualization and were not used for statistical inference.

**2 Supplementary Tables**

**Supplementary Table S1A.** Guideline corpus (anchor documents).

| **ID** | **Document** | **PMID** |
| --- | --- | --- |
| CN1 | Clinical Guidelines on Compression Therapy in Venous Diseases | 39032593 |
| INT1 | SVS/AVF/AVLS clinical practice guidelines for management of varicose veins (Part I) | 36326210 |
| INT2 | SVS/AVF/AVLS clinical practice guidelines for management of varicose veins (Part II) | 37652254 |
| INT3 | ESVS 2022 Clinical Practice Guidelines on Management of Chronic Venous Disease of the Lower Limbs | 35027279 |
| INT4 | SCAI 2025 Clinical Practice Guidelines for Management of Chronic Venous Disease | 41019905 |
| INT5 | NICE CG168: diagnosis and management of varicose veins in the legs (summary of guidance) | 23884969 |

**Supplementary Table S1B.** Patient-facing question set and a priori guideline mapping.

| **Q#** | **Patient-facing question (English)** | **Domain (5×4)** | **China guideline anchor (CN)** | **International guideline anchor (INT)** |
| --- | --- | --- | --- | --- |
| 1 | What are varicose veins? | Basics & prognosis | - | INT3 |
| 2 | What causes varicose veins? | Basics & prognosis | - | INT3 |
| 3 | Who is at higher risk of developing varicose veins? | Basics & prognosis | - | INT3 |
| 4 | Can varicose veins worsen over time without treatment? | Basics & prognosis | - | INT3 |
| 5 | Which symptoms of varicose veins require urgent medical care? | Assessment & diagnosis | - | INT5 |
| 6 | Should I get a duplex ultrasound to evaluate varicose veins? | Assessment & diagnosis | - | INT1 |
| 7 | How are varicose veins staged using the Clinical–Etiology–Anatomy–Pathophysiology classification? | Assessment & diagnosis | - | INT3 |
| 8 | How can a clinician confirm that my symptoms are due to varicose veins rather than artery disease? | Assessment & diagnosis | - | INT3 |
| 9 | What lifestyle changes help symptoms from varicose veins? | Conservative care & compression | - | INT3 |
| 10 | Can exercise improve symptoms from varicose veins? | Conservative care & compression | - | INT3 |
| 11 | What compression stocking pressure is recommended for varicose veins? | Conservative care & compression | CN1 | INT2 |
| 12 | When should compression stockings not be used for varicose veins? | Conservative care & compression | CN1 | INT2 |
| 13 | What is endovenous laser ablation for varicose veins? | Interventions & aftercare | - | INT1 |
| 14 | What is endovenous radiofrequency ablation for varicose veins? | Interventions & aftercare | - | INT1 |
| 15 | What is ultrasound-guided foam sclerotherapy for varicose veins? | Interventions & aftercare | - | INT1 |
| 16 | What should I do immediately after a varicose vein procedure? | Interventions & aftercare | - | INT2 |
| 17 | How can blood clots be prevented after varicose vein procedures? | Complications & long-term | - | INT2 |
| 18 | How are skin changes caused by varicose veins treated? | Complications & long-term | CN1 | INT3 |
| 19 | How are venous leg ulcers related to varicose veins treated? | Complications & long-term | CN1 | INT4 |
| 20 | Can varicose veins come back after treatment? | Complications & long-term | - | INT3 |

Abbreviations: CN = China guideline anchor; INT = international guideline anchor. Domains were prespecified across the varicose vein care pathway (five domains x four subdomains).

**Supplementary Table S1C.** Top 50 varicose-vein-related public queries from Google Trends over the past 5 years

Source fields are shown as provided in the Google Trends export. Search-interest values are relative values within the source list.

| **Rank** | **Google Trends query** | **Search interest** | **Increase percent** | **Theme** | **Matched benchmark question(s)** | **Crosswalk status** |
| --- | --- | --- | --- | --- | --- | --- |
| 1 | varicose veins treatment | 100 | 0.06 | Treatment options (generic) | Q13–Q15 | Thematic |
| 2 | varicose vein | 59 | -0.07 | Definition / disease explanation | Q1 | Direct |
| 3 | varicose veins legs | 44 | -0.03 | Symptoms / manifestations | Q5 | Thematic |
| 4 | varicose veins causes | 35 | 0.04 | Cause / etiology | Q2 | Direct |
| 5 | what is varicose veins | 32 | 0.4 | Definition / disease explanation | Q1 | Direct |
| 6 | varicose veins pain | 32 | -0.1 | Symptoms / manifestations | Q5 | Thematic |
| 7 | varicose veins cause | 29 | 0.03 | Cause / etiology | Q2 | Direct |
| 8 | treatment for varicose veins | 27 | 0.02 | Treatment options (generic) | Q13–Q15 | Thematic |
| 9 | varicose veins symptoms | 26 | 0.3 | Symptoms / manifestations | Q5 | Thematic |
| 10 | veins in legs | 24 | 0.07 | Symptoms / manifestations | Q5 | Thematic |
| 11 | varicose veins in legs | 23 | 0.02 | Symptoms / manifestations | Q5 | Thematic |
| 12 | varicose veins pregnancy | 23 | -0.3 | Risk factor context (pregnancy) | Q3 | Thematic |
| 13 | spider veins | 21 | -0.2 | Related but distinct condition | No clear direct benchmark item | No clear match |
| 14 | varicose veins stockings | 20 | 0.2 | Compression therapy | Q11 | Thematic |
| 15 | varicose veins surgery | 20 | -0.08 | Treatment options (generic) | Q13–Q15 | Thematic |
| 16 | varicose veins meaning | 20 | 0.6 | Definition / disease explanation | Q1 | Direct |
| 17 | what causes varicose veins | 19 | 0.05 | Cause / etiology | Q2 | Direct |
| 18 | varicose veins cream | 18 | -0.02 | Medication / topical treatment | No clear direct benchmark item | No clear match |
| 19 | varicose veins socks | 18 | 0.3 | Compression therapy | Q11 | Thematic |
| 20 | varicose veins doctor | 16 | 0.5 | Seeking medical evaluation | Q5 | Thematic |
| 21 | what are varicose veins | 16 | 0.1 | Definition / disease explanation | Q1 | Direct |
| 22 | get rid of varicose veins | 15 | -0.2 | Treatment / cure-seeking | Q20 | Thematic |
| 23 | varicose vein treatment | 15 | -0.2 | Treatment options (generic) | Q13–Q15 | Thematic |
| 24 | icd 10 varicose veins | 14 | -0.08 | Administrative / language-specific query | No clear direct benchmark item | No clear match |
| 25 | varicose veins icd 10 | 13 | -0.08 | Administrative / language-specific query | No clear direct benchmark item | No clear match |
| 26 | compression socks | 13 | 0.4 | Compression therapy | Q11 | Thematic |
| 27 | treatment of varicose veins | 13 | 0.04 | Treatment options (generic) | Q13–Q15 | Thematic |
| 28 | stockings for varicose veins | 13 | 0.2 | Compression therapy | Q11 | Thematic |
| 29 | varicose veins compression stockings | 12 | 0.2 | Compression therapy | Q11 | Thematic |
| 30 | compression stockings | 11 | 0.1 | Compression therapy | Q11 | Thematic |
| 31 | how to get rid of varicose veins | 10 | -0.2 | Treatment / cure-seeking | Q20 | Thematic |
| 32 | prevent varicose veins | 10 | -0.07 | Prevention / self-management | Q9 | Thematic |
| 33 | vericose veins | 9 | -0.03 | Definition / disease explanation | Q1 | Direct |
| 34 | varicose veins medicine | 9 | 0.3 | Medication / topical treatment | No clear direct benchmark item | No clear match |
| 35 | varicose veins in pregnancy | 9 | -0.2 | Risk factor context (pregnancy) | Q3 | Thematic |
| 36 | socks for varicose veins | 9 | 0.2 | Compression therapy | Q11 | Thematic |
| 37 | varicose veins exercise | 9 | 0.1 | Exercise | Q10 | Direct |
| 38 | cause of varicose veins | 9 | 0.1 | Cause / etiology | Q2 | Direct |
| 39 | sclerotherapy | 9 | 0.2 | Sclerotherapy | Q15 | Direct |
| 40 | varicose veins laser treatment | 8 | -0.05 | Laser treatment | Q13 | Direct |
| 41 | compression stockings for varicose veins | 8 | 0.3 | Compression therapy | Q11 | Thematic |
| 42 | causes of varicose veins | 8 | 0.02 | Cause / etiology | Q2 | Direct |
| 43 | symptoms of varicose veins | 8 | -0.04 | Symptoms / manifestations | Q5 | Thematic |
| 44 | varicose veins in hindi | 7 | 0.7 | Administrative / language-specific query | No clear direct benchmark item | No clear match |
| 45 | varicose veins removal | 7 | -0.3 | Treatment / cure-seeking | Q20 | Thematic |
| 46 | painful varicose veins | 7 | -0.3 | Symptoms / manifestations | Q5 | Thematic |
| 47 | dvt | 7 | 0.2 | Thrombosis concern | Q17 | Thematic |
| 48 | cream for varicose veins | 7 | 0.04 | Medication / topical treatment | No clear direct benchmark item | No clear match |
| 49 | varicose veins treatment cost | 7 | 0.07 | Treatment options (generic) | Q13–Q15 | Thematic |
| 50 | compression socks for varicose veins | 7 | 0.3 | Compression therapy | Q11 | Thematic |

**Supplementary Table S1D.** Top 50 varicose-vein-related public queries from Baidu Zhidao over the past 5 years, with English translations

Chinese queries are reproduced as supplied in the source file. English translations are provided for cross-source comparison and supplementary interpretation.

| **Rank** | **Original Chinese query** | **English translation** | **Theme** | **Matched benchmark question(s)** | **Crosswalk status** |
| --- | --- | --- | --- | --- | --- |
| 1 | 孩子下肢静脉曲张:预防为先 | Prevention first for lower-extremity varicose veins in children | Risk factor context | Q3 | Thematic |
| 2 | 妊娠后期静脉曲张的预防和治疗 | Prevention and treatment of varicose veins in late pregnancy | Risk factor context | Q3 | Thematic |
| 3 | 什么是静脉曲张 | What are varicose veins? | Definition / disease explanation | Q1 | Direct |
| 4 | 静脉曲张是怎么回事啊 | What is going on with varicose veins? | Definition / disease explanation | Q1 | Direct |
| 5 | 静脉曲张是什么意思? | What does varicose veins mean? | Definition / disease explanation | Q1 | Direct |
| 6 | 什么是静脉曲张 | What are varicose veins? | Definition / disease explanation | Q1 | Direct |
| 7 | 流水线长时间站立对身体有什么危害? | What harm can prolonged standing on an assembly line do to the body? | Risk factor context | Q3 | Thematic |
| 8 | 静脉曲张怎么治疗最好 | What is the best treatment for varicose veins? | Treatment options (generic) | Q13–Q15 / Q20 | Thematic |
| 9 | 如何治愈静脉曲张? | How can varicose veins be cured? | Treatment options (generic) | Q13–Q15 / Q20 | Thematic |
| 10 | 什么是静脉曲张 | What are varicose veins? | Definition / disease explanation | Q1 | Direct |
| 11 | 什么是静脉曲张 | What are varicose veins? | Definition / disease explanation | Q1 | Direct |
| 12 | 静脉曲张是什么病? | What kind of disease are varicose veins? | Definition / disease explanation | Q1 | Direct |
| 13 | 静脉曲张的原因 – 静脉曲张有什么症状 – 静脉曲张注意事项 | Causes of varicose veins – symptoms of varicose veins – precautions for varicose veins | Cause / etiology | Q2 | Direct |
| 14 | 什么是静脉曲张 | What are varicose veins? | Definition / disease explanation | Q1 | Direct |
| 15 | 精索静脉曲张 | Varicocele | Related but distinct condition | No clear direct benchmark item | No clear match |
| 16 | 静脉曲张的十大危害 | The ten major harms of varicose veins | Progression / harms | Q4 | Thematic |
| 17 | 静脉曲张的症状与治疗 | Symptoms and treatment of varicose veins | Symptoms / manifestations | Q5 | Thematic |
| 18 | 静脉曲张严重吗?看完你就懂了 | Are varicose veins serious? You will understand after reading this | Progression / harms | Q4 | Thematic |
| 19 | 静脉曲张的一些特点 | Some characteristics of varicose veins | General disease information | Q1 / Q4 / Q5 | Thematic |
| 20 | 静脉曲张的一些特点 | Some characteristics of varicose veins | General disease information | Q1 / Q4 / Q5 | Thematic |
| 21 | 静脉曲张是怎么引起的 | What causes varicose veins? | Cause / etiology | Q2 | Direct |
| 22 | 什么叫静脉曲张? | What are varicose veins? | Definition / disease explanation | Q1 | Direct |
| 23 | 静脉曲张四大危害 | The four major harms of varicose veins | Progression / harms | Q4 | Thematic |
| 24 | 静脉曲张有哪些表现? | What manifestations do varicose veins have? | Symptoms / manifestations | Q5 | Thematic |
| 25 | 静脉曲张的表现有哪些 | What are the manifestations of varicose veins? | Symptoms / manifestations | Q5 | Thematic |
| 26 | 静脉曲张的危害到底有多大 | How harmful are varicose veins? | Progression / harms | Q4 | Thematic |
| 27 | 静脉曲张的危害有哪些 | What are the harms of varicose veins? | Progression / harms | Q4 | Thematic |
| 28 | 静脉曲张到底是什么病 | What disease exactly are varicose veins? | Definition / disease explanation | Q1 | Direct |
| 29 | 静脉曲张的症状有哪些 | What are the symptoms of varicose veins? | Symptoms / manifestations | Q5 | Thematic |
| 30 | 静脉曲张是什么 | What are varicose veins? | Definition / disease explanation | Q1 | Direct |
| 31 | 静脉曲张怎么回事 | What is going on with varicose veins? | Definition / disease explanation | Q1 | Direct |
| 32 | 什么是静脉曲张? | What are varicose veins? | Definition / disease explanation | Q1 | Direct |
| 33 | 静脉曲张应该如何缓解? | How should varicose veins be relieved? | Lifestyle / self-management | Q9 | Thematic |
| 34 | 腿上的静脉曲张怎样才能治好 | How can varicose veins on the legs be cured? | Treatment options (generic) | Q13–Q15 / Q20 | Thematic |
| 35 | 静脉曲张你必须知道的3点! | Three things you must know about varicose veins! | General disease information | Q1 / Q4 / Q5 | Thematic |
| 36 | 静脉曲张的治疗方法 | Treatment methods for varicose veins | Treatment options (generic) | Q13–Q15 / Q20 | Thematic |
| 37 | 什么是静脉曲张症状原来这9种都是 | What are varicose veins symptoms? It turns out these 9 are all symptoms | Definition / disease explanation | Q1 | Direct |
| 38 | 什么是静脉曲张 静脉曲张是怎么回事 | What are varicose veins? What is going on with varicose veins? | Definition / disease explanation | Q1 | Direct |
| 39 | 静脉曲张的危害是什么 | What are the harms of varicose veins? | Progression / harms | Q4 | Thematic |
| 40 | 哪些原因会导致静脉曲张? | What causes varicose veins? | Cause / etiology | Q2 | Direct |
| 41 | 静脉曲张锻炼方法 静脉曲张有哪些锻炼方法 | Exercise methods for varicose veins; what exercises help varicose veins? | Exercise | Q10 | Direct |
| 42 | 静脉曲张治疗如何选择治疗方法 | How should treatment methods be selected for varicose veins? | Treatment options (generic) | Q13–Q15 / Q20 | Thematic |
| 43 | 治疗静脉曲张的办法有哪些,比较管用的 | What are the ways to treat varicose veins, and which are more effective? | Other | No clear direct benchmark item | No clear match |
| 44 | 如何治疗静脉曲张,静脉曲张治疗方法 | How to treat varicose veins; treatment methods for varicose veins | Treatment options (generic) | Q13–Q15 / Q20 | Thematic |
| 45 | 静脉曲张要怎么治疗?下肢静脉曲张能治好吗?拜托了各位 谢谢 | How should varicose veins be treated? Can lower-extremity varicose veins be cured? | Treatment options (generic) | Q13–Q15 / Q20 | Thematic |
| 46 | 是静脉曲张吗 | Is this varicose veins? | Symptoms / manifestations | Q5 | Thematic |
| 47 | 静脉曲张是如何引起的 | How are varicose veins caused? | Cause / etiology | Q2 | Direct |
| 48 | 静脉曲张是怎么回事 | What is going on with varicose veins? | Definition / disease explanation | Q1 | Direct |
| 49 | 静脉曲张如何保健? | How should varicose veins be managed for health maintenance? | Lifestyle / self-management | Q9 | Thematic |
| 50 | 什么原因引起全身静脉曲张 | What causes systemic varicose veins? | Cause / etiology | Q2 | Direct |

**Supplementary Table S1E.** Crosswalk between the 20-question benchmark and public query-demand proxies from Google Trends and Baidu Zhidao

Definitions: Direct = near-synonymous public query or explicit patient-facing query matching the same intent; Thematic = same general domain but different specificity or framing; No clear match = no comparable high-frequency public query identifiable in the top lists.

| **No.** | **Benchmark question** | **Matched Google Trends queries** | **Matched Baidu Zhidao queries (English)** | **Crosswalk status** | **Interpretive note** |
| --- | --- | --- | --- | --- | --- |
| 1 | What are varicose veins? | what is varicose veins; what are varicose veins; varicose veins meaning | 什么是静脉曲张; 静脉曲张是什么意思?; 什么叫静脉曲张? | Direct | High overlap for basic disease-definition intent. |
| 2 | What causes varicose veins? | varicose veins causes; what causes varicose veins; cause of varicose veins | 静脉曲张是怎么引起的; 哪些原因会导致静脉曲张?; 静脉曲张是如何引起的 | Direct | High overlap for cause/etiology intent. |
| 3 | Who is at higher risk of developing varicose veins? | varicose veins pregnancy; varicose veins in pregnancy | 孩子下肢静脉曲张:预防为先; 妊娠后期静脉曲张的预防和治疗; 流水线长时间站立对身体有什么危害? | Thematic | Public queries emphasize concrete risk contexts rather than a formal risk-factor question. |
| 4 | Can varicose veins worsen over time without treatment? | — | 静脉曲张的十大危害; 静脉曲张严重吗?; 静脉曲张的危害到底有多大 | Thematic | Progression is expressed in public queries as severity/harms rather than untreated natural history. |
| 5 | Which symptoms of varicose veins require urgent medical care? | varicose veins symptoms; symptoms of varicose veins; varicose veins pain | 静脉曲张有哪些表现?; 静脉曲张的症状有哪些; 什么是静脉曲张症状原来这9种都是 | Thematic | Public interest covers symptoms, but not specifically red-flag or urgent symptoms. |
| 6 | Should I get a duplex ultrasound to evaluate varicose veins? | — | — | No clear match | Duplex ultrasound is guideline-important but not visible in top public query lists. |
| 7 | How are varicose veins staged using the Clinical–Etiology–Anatomy–Pathophysiology classification? | — | — | No clear match | CEAP staging is highly clinical and absent from public query proxies. |
| 8 | How can a clinician confirm that my symptoms are due to varicose veins rather than artery disease? | — | — | No clear match | Differentiation from arterial disease is clinically important but not directly reflected in public query lists. |
| 9 | What lifestyle changes help symptoms from varicose veins? | prevent varicose veins; get rid of varicose veins | 静脉曲张应该如何缓解?; 静脉曲张如何保健? | Thematic | Public queries ask about symptom relief and self-management in less technical language. |
| 10 | Can exercise improve symptoms from varicose veins? | varicose veins exercise | 静脉曲张锻炼方法 静脉曲张有哪些锻炼方法 | Direct | Direct overlap for exercise-related self-management. |
| 11 | What compression stocking pressure is recommended for varicose veins? | varicose veins stockings; compression socks; stockings for varicose veins | — | Thematic | Compression garments are a common public topic, but pressure levels are not explicitly queried. |
| 12 | When should compression stockings not be used for varicose veins? | — | — | No clear match | Contraindications to compression are guideline-important but not represented in the top query lists. |
| 13 | What is endovenous laser ablation for varicose veins? | varicose veins laser treatment | — | Direct | Direct overlap for laser-based treatment intent. |
| 14 | What is endovenous radiofrequency ablation for varicose veins? | — | — | No clear match | Radiofrequency ablation is not captured in the top public query lists. |
| 15 | What is ultrasound-guided foam sclerotherapy for varicose veins? | sclerotherapy | — | Direct | Direct overlap for sclerotherapy-based treatment intent. |
| 16 | What should I do immediately after a varicose vein procedure? | — | — | No clear match | Immediate post-procedure care is absent from the public query lists. |
| 17 | How can blood clots be prevented after varicose vein procedures? | dvt | — | Thematic | Public concern about thrombosis is visible, but not framed as post-procedural clot prevention. |
| 18 | How are skin changes caused by varicose veins treated? | — | — | No clear match | Skin changes are clinically relevant downstream manifestations but do not appear as a distinct public query theme. |
| 19 | How are venous leg ulcers related to varicose veins treated? | — | — | No clear match | Venous leg ulcers are not explicitly represented in the top public query lists. |
| 20 | Can varicose veins come back after treatment? | get rid of varicose veins; varicose veins removal | 如何治愈静脉曲张?; 腿上的静脉曲张怎样才能治好; 下肢静脉曲张能治好吗? | Thematic | Public queries emphasize cure/removal, which partially overlaps with durability/recurrence concerns. |

Abbreviation: CEAP, Clinical–Etiology–Anatomy–Pathophysiology.

**Supplementary Table S2.** Pairwise between-model comparisons (Wilcoxon signed-rank; paired by question; Holm adjustment within outcome across 10 model pairs).

| **Outcome** | **Comparison** | **Wilcoxon statistic** | **Raw P value** | **Holm-adjusted P value** |
| --- | --- | --- | --- | --- |
| DISCERN total score (16-80) | ChatGPT 5.2 vs DeepSeek-V3.2 | 67.500 | 0.1779 | 1.0000 |
| DISCERN total score (16-80) | ChatGPT 5.2 vs Gemini 3 Pro | 74.500 | 0.2775 | 1.0000 |
| DISCERN total score (16-80) | ChatGPT 5.2 vs Grok 4.1 | 101.000 | 0.9255 | 1.0000 |
| DISCERN total score (16-80) | ChatGPT 5.2 vs Qwen3-Max | 61.500 | 0.1071 | 0.7497 |
| DISCERN total score (16-80) | DeepSeek-V3.2 vs Gemini 3 Pro | 39.000 | 0.0142 | 0.1277 |
| DISCERN total score (16-80) | DeepSeek-V3.2 vs Grok 4.1 | 72.000 | 0.2218 | 1.0000 |
| DISCERN total score (16-80) | DeepSeek-V3.2 vs Qwen3-Max | 37.500 | 0.0107 | 0.1069 |
| DISCERN total score (16-80) | Gemini 3 Pro vs Grok 4.1 | 71.500 | 0.2168 | 1.0000 |
| DISCERN total score (16-80) | Gemini 3 Pro vs Qwen3-Max | 80.000 | 0.3584 | 1.0000 |
| DISCERN total score (16-80) | Grok 4.1 vs Qwen3-Max | 55.500 | 0.0667 | 0.5335 |
| EQIP (% Yes items) | ChatGPT 5.2 vs DeepSeek-V3.2 | 17.500 | 0.0246 | 0.2464 |
| EQIP (% Yes items) | ChatGPT 5.2 vs Gemini 3 Pro | 85.500 | 0.9534 | 1.0000 |
| EQIP (% Yes items) | ChatGPT 5.2 vs Grok 4.1 | 78.000 | 0.3967 | 1.0000 |
| EQIP (% Yes items) | ChatGPT 5.2 vs Qwen3-Max | 81.000 | 0.8147 | 1.0000 |
| EQIP (% Yes items) | DeepSeek-V3.2 vs Gemini 3 Pro | 62.000 | 0.2144 | 1.0000 |
| EQIP (% Yes items) | DeepSeek-V3.2 vs Grok 4.1 | 64.000 | 0.2967 | 1.0000 |
| EQIP (% Yes items) | DeepSeek-V3.2 vs Qwen3-Max | 52.000 | 0.0801 | 0.7213 |
| EQIP (% Yes items) | Gemini 3 Pro vs Grok 4.1 | 87.500 | 0.6354 | 1.0000 |
| EQIP (% Yes items) | Gemini 3 Pro vs Qwen3-Max | 80.500 | 0.8004 | 1.0000 |
| EQIP (% Yes items) | Grok 4.1 vs Qwen3-Max | 71.500 | 0.3756 | 1.0000 |
| JAMA benchmark (0-4) | ChatGPT 5.2 vs DeepSeek-V3.2 | 0.000 | 0.1573 | 1.0000 |
| JAMA benchmark (0-4) | ChatGPT 5.2 vs Gemini 3 Pro | 0.000 | 0.1573 | 1.0000 |
| JAMA benchmark (0-4) | ChatGPT 5.2 vs Grok 4.1 | 0.000 | 0.0833 | 0.6661 |
| JAMA benchmark (0-4) | ChatGPT 5.2 vs Qwen3-Max | 19.500 | 1.0000 | 1.0000 |
| JAMA benchmark (0-4) | DeepSeek-V3.2 vs Gemini 3 Pro | 0.000 | 1.0000 | 1.0000 |
| JAMA benchmark (0-4) | DeepSeek-V3.2 vs Grok 4.1 | 0.000 | 0.0253 | 0.2535 |
| JAMA benchmark (0-4) | DeepSeek-V3.2 vs Qwen3-Max | 0.000 | 0.1573 | 1.0000 |
| JAMA benchmark (0-4) | Gemini 3 Pro vs Grok 4.1 | 0.000 | 0.0253 | 0.2535 |
| JAMA benchmark (0-4) | Gemini 3 Pro vs Qwen3-Max | 0.000 | 0.1573 | 1.0000 |
| JAMA benchmark (0-4) | Grok 4.1 vs Qwen3-Max | 18.000 | 0.1797 | 1.0000 |
| GQS (1-5) | ChatGPT 5.2 vs DeepSeek-V3.2 | 29.000 | 0.0497 | 0.4471 |
| GQS (1-5) | ChatGPT 5.2 vs Gemini 3 Pro | 34.000 | 0.2568 | 1.0000 |
| GQS (1-5) | ChatGPT 5.2 vs Grok 4.1 | 48.000 | 0.4419 | 1.0000 |
| GQS (1-5) | ChatGPT 5.2 vs Qwen3-Max | 66.000 | 1.0000 | 1.0000 |
| GQS (1-5) | DeepSeek-V3.2 vs Gemini 3 Pro | 32.000 | 0.2234 | 1.0000 |
| GQS (1-5) | DeepSeek-V3.2 vs Grok 4.1 | 17.000 | 0.0588 | 0.4703 |
| GQS (1-5) | DeepSeek-V3.2 vs Qwen3-Max | 0.000 | 0.0083 | 0.0832 |
| GQS (1-5) | Gemini 3 Pro vs Grok 4.1 | 66.000 | 1.0000 | 1.0000 |
| GQS (1-5) | Gemini 3 Pro vs Qwen3-Max | 34.000 | 0.2568 | 1.0000 |
| GQS (1-5) | Grok 4.1 vs Qwen3-Max | 18.000 | 0.1797 | 1.0000 |
| Automated Readability Index (ARI) | ChatGPT 5.2 vs DeepSeek-V3.2 | 102.500 | 0.9563 | 1.0000 |
| Automated Readability Index (ARI) | ChatGPT 5.2 vs Gemini 3 Pro | 89.000 | 0.5706 | 1.0000 |
| Automated Readability Index (ARI) | ChatGPT 5.2 vs Grok 4.1 | 24.000 | 0.0014 | 0.0086 |
| Automated Readability Index (ARI) | ChatGPT 5.2 vs Qwen3-Max | 42.000 | 0.0172 | 0.0859 |
| Automated Readability Index (ARI) | DeepSeek-V3.2 vs Gemini 3 Pro | 86.000 | 0.4980 | 1.0000 |
| Automated Readability Index (ARI) | DeepSeek-V3.2 vs Grok 4.1 | 20.000 | 0.0007 | 0.0050 |
| Automated Readability Index (ARI) | DeepSeek-V3.2 vs Qwen3-Max | 17.000 | 0.0004 | 0.0032 |
| Automated Readability Index (ARI) | Gemini 3 Pro vs Grok 4.1 | 15.000 | 0.0003 | 0.0024 |
| Automated Readability Index (ARI) | Gemini 3 Pro vs Qwen3-Max | 10.000 | 0.0001 | 0.0008 |
| Automated Readability Index (ARI) | Grok 4.1 vs Qwen3-Max | 82.000 | 0.4091 | 1.0000 |
| Flesch Reading Ease Score (FRES) | ChatGPT 5.2 vs DeepSeek-V3.2 | 72.000 | 0.2247 | 0.4495 |
| Flesch Reading Ease Score (FRES) | ChatGPT 5.2 vs Gemini 3 Pro | 80.500 | 0.3698 | 0.4495 |
| Flesch Reading Ease Score (FRES) | ChatGPT 5.2 vs Grok 4.1 | 4.500 | 0.0002 | 0.0013 |
| Flesch Reading Ease Score (FRES) | ChatGPT 5.2 vs Qwen3-Max | 0.000 | <0.0001 | <0.0001 |
| Flesch Reading Ease Score (FRES) | DeepSeek-V3.2 vs Gemini 3 Pro | 32.500 | 0.0071 | 0.0213 |
| Flesch Reading Ease Score (FRES) | DeepSeek-V3.2 vs Grok 4.1 | 16.000 | 0.0011 | 0.0054 |
| Flesch Reading Ease Score (FRES) | DeepSeek-V3.2 vs Qwen3-Max | 0.000 | <0.0001 | <0.0001 |
| Flesch Reading Ease Score (FRES) | Gemini 3 Pro vs Grok 4.1 | 7.000 | 0.0003 | 0.0016 |
| Flesch Reading Ease Score (FRES) | Gemini 3 Pro vs Qwen3-Max | 0.000 | <0.0001 | <0.0001 |
| Flesch Reading Ease Score (FRES) | Grok 4.1 vs Qwen3-Max | 18.000 | 0.0012 | 0.0054 |
| Gunning Fog Index (GFI) | ChatGPT 5.2 vs DeepSeek-V3.2 | 49.500 | 0.0399 | 0.1597 |
| Gunning Fog Index (GFI) | ChatGPT 5.2 vs Gemini 3 Pro | 86.000 | 0.4980 | 0.4980 |
| Gunning Fog Index (GFI) | ChatGPT 5.2 vs Grok 4.1 | 20.000 | 0.0007 | 0.0050 |
| Gunning Fog Index (GFI) | ChatGPT 5.2 vs Qwen3-Max | 0.000 | <0.0001 | <0.0001 |
| Gunning Fog Index (GFI) | DeepSeek-V3.2 vs Gemini 3 Pro | 49.500 | 0.0399 | 0.1597 |
| Gunning Fog Index (GFI) | DeepSeek-V3.2 vs Grok 4.1 | 60.000 | 0.0973 | 0.1946 |
| Gunning Fog Index (GFI) | DeepSeek-V3.2 vs Qwen3-Max | 1.000 | <0.0001 | <0.0001 |
| Gunning Fog Index (GFI) | Gemini 3 Pro vs Grok 4.1 | 38.500 | 0.0121 | 0.0604 |
| Gunning Fog Index (GFI) | Gemini 3 Pro vs Qwen3-Max | 2.000 | <0.0001 | <0.0001 |
| Gunning Fog Index (GFI) | Grok 4.1 vs Qwen3-Max | 21.000 | 0.0009 | 0.0051 |
| Flesch-Kincaid Grade Level (FKGL) | ChatGPT 5.2 vs DeepSeek-V3.2 | 77.000 | 0.3118 | 1.0000 |
| Flesch-Kincaid Grade Level (FKGL) | ChatGPT 5.2 vs Gemini 3 Pro | 86.000 | 0.4980 | 1.0000 |
| Flesch-Kincaid Grade Level (FKGL) | ChatGPT 5.2 vs Grok 4.1 | 12.000 | 0.0001 | 0.0011 |
| Flesch-Kincaid Grade Level (FKGL) | ChatGPT 5.2 vs Qwen3-Max | 14.000 | 0.0002 | 0.0015 |
| Flesch-Kincaid Grade Level (FKGL) | DeepSeek-V3.2 vs Gemini 3 Pro | 101.000 | 0.8983 | 1.0000 |
| Flesch-Kincaid Grade Level (FKGL) | DeepSeek-V3.2 vs Grok 4.1 | 23.000 | 0.0012 | 0.0073 |
| Flesch-Kincaid Grade Level (FKGL) | DeepSeek-V3.2 vs Qwen3-Max | 11.000 | 0.0001 | 0.0009 |
| Flesch-Kincaid Grade Level (FKGL) | Gemini 3 Pro vs Grok 4.1 | 22.500 | 0.0012 | 0.0073 |
| Flesch-Kincaid Grade Level (FKGL) | Gemini 3 Pro vs Qwen3-Max | 10.000 | 0.0001 | 0.0008 |
| Flesch-Kincaid Grade Level (FKGL) | Grok 4.1 vs Qwen3-Max | 99.000 | 0.8408 | 1.0000 |
| Coleman-Liau Index (CL) | ChatGPT 5.2 vs DeepSeek-V3.2 | 97.000 | 0.7841 | 0.7841 |
| Coleman-Liau Index (CL) | ChatGPT 5.2 vs Gemini 3 Pro | 46.000 | 0.0266 | 0.0533 |
| Coleman-Liau Index (CL) | ChatGPT 5.2 vs Grok 4.1 | 7.000 | <0.0001 | 0.0002 |
| Coleman-Liau Index (CL) | ChatGPT 5.2 vs Qwen3-Max | 0.000 | <0.0001 | <0.0001 |
| Coleman-Liau Index (CL) | DeepSeek-V3.2 vs Gemini 3 Pro | 7.000 | <0.0001 | 0.0002 |
| Coleman-Liau Index (CL) | DeepSeek-V3.2 vs Grok 4.1 | 6.000 | <0.0001 | 0.0002 |
| Coleman-Liau Index (CL) | DeepSeek-V3.2 vs Qwen3-Max | 0.000 | <0.0001 | <0.0001 |
| Coleman-Liau Index (CL) | Gemini 3 Pro vs Grok 4.1 | 0.000 | <0.0001 | <0.0001 |
| Coleman-Liau Index (CL) | Gemini 3 Pro vs Qwen3-Max | 0.000 | <0.0001 | <0.0001 |
| Coleman-Liau Index (CL) | Grok 4.1 vs Qwen3-Max | 9.500 | 0.0001 | 0.0002 |
| SMOG | ChatGPT 5.2 vs DeepSeek-V3.2 | 58.000 | 0.0826 | 0.3302 |
| SMOG | ChatGPT 5.2 vs Gemini 3 Pro | 72.000 | 0.2305 | 0.6915 |
| SMOG | ChatGPT 5.2 vs Grok 4.1 | 15.000 | 0.0003 | 0.0026 |
| SMOG | ChatGPT 5.2 vs Qwen3-Max | 35.000 | 0.0073 | 0.0584 |
| SMOG | DeepSeek-V3.2 vs Gemini 3 Pro | 81.000 | 0.3884 | 0.7768 |
| SMOG | DeepSeek-V3.2 vs Grok 4.1 | 52.500 | 0.0532 | 0.2658 |
| SMOG | DeepSeek-V3.2 vs Qwen3-Max | 45.500 | 0.0266 | 0.1599 |
| SMOG | Gemini 3 Pro vs Grok 4.1 | 37.000 | 0.0094 | 0.0660 |
| SMOG | Gemini 3 Pro vs Qwen3-Max | 27.000 | 0.0023 | 0.0209 |
| SMOG | Grok 4.1 vs Qwen3-Max | 105.000 | 1.0000 | 1.0000 |

Notes: P values are two-sided. Analyses are paired by question (n = 20). Holm adjustment is applied within each outcome across the 10 pairwise comparisons.

**Supplementary Table S3.** Response length (word count) across models (n = 20 questions per model).

| Model | Mean ± SD word count | Median [Q1, Q3] | Range |
| --- | --- | --- | --- |
| ChatGPT 5.2 | 292.20 ± 97.17 | 302.00 [206.75, 364.50] | 120–466 |
| DeepSeek-V3.2 | 554.75 ± 83.73 | 569.00 [504.50, 620.25] | 379–673 |
| Gemini 3 Pro | 423.45 ± 83.14 | 421.00 [385.25, 480.50] | 225–572 |
| Grok 4.1 | 268.60 ± 68.62 | 269.50 [235.00, 307.50] | 119–391 |
| Qwen3-Max | 339.35 ± 54.01 | 345.50 [298.25, 376.50] | 241–454 |

Notes: Word count was calculated on the plain-text response after removal of non-content interface artifacts only; URLs and bullet formatting, when present, were retained as text. Values are summarized across 20 questions per model.
